# Nanopore-Based Target Sequence Detection

**DOI:** 10.1371/journal.pone.0154426

**Published:** 2016-05-05

**Authors:** Trevor J. Morin, Tyler Shropshire, Xu Liu, Kyle Briggs, Cindy Huynh, Vincent Tabard-Cossa, Hongyun Wang, William B. Dunbar

**Affiliations:** 1 Two Pore Guys Inc., Santa Cruz, CA, United States of America; 2 Department of Physics, University of Ottawa, Ontario, Canada; 3 Baskin School of Engineering, University of California Santa Cruz, Santa Cruz, CA, United States of America; Northeastern University, UNITED STATES

## Abstract

The promise of portable diagnostic devices relies on three basic requirements: comparable sensitivity to established platforms, inexpensive manufacturing and cost of operations, and the ability to survive rugged field conditions. Solid state nanopores can meet all these requirements, but to achieve high manufacturing yields at low costs, assays must be tolerant to fabrication imperfections and to nanopore enlargement during operation. This paper presents a model for molecular engineering techniques that meets these goals with the aim of detecting target sequences within DNA. In contrast to methods that require precise geometries, we demonstrate detection using a range of pore geometries. As a result, our assay model tolerates any pore-forming method and in-situ pore enlargement. Using peptide nucleic acid (PNA) probes modified for conjugation with synthetic bulk-adding molecules, pores ranging 15-50 nm in diameter are shown to detect individual PNA-bound DNA. Detection of the CFTRΔF508 gene mutation, a codon deletion responsible for ∼66% of all cystic fibrosis chromosomes, is demonstrated with a 26-36 nm pore size range by using a size-enhanced PNA probe. A mathematical framework for assessing the statistical significance of detection is also presented.

## Introduction

Nucleic acid diagnostics is a billion dollar industry that is expected to double in revenue in the next 5 years, with growth catalyzed by next-generation sequencing technologies that can efficiently sequence entire genomes or large portions of genomes [1]. With these technologies, previously unknown species and genotypes are being uncovered [2–4], new disease related genomic mutations are being discovered [5], and a multitude of allelic differences between two individuals can be efficiently compared [6, 7]. Once the initial comprehensive sequencing of an organism is performed and regions of interest are identified, there are many applications for which it is no longer necessary to re-sequence the genome. Instead, it is more economical in both time and cost to test for the presence of only a specific target sequence or group of sequences. As an example, once a gene mutation has been identified from full exome sequencing as the cause for a familial inherited disease, additional family members only need to test for the disease-causing sequence, without needing to sequence their full exomes. This is the case for familial diabetes [8], Alzheimer’s disease [9], and breast cancer [10], among other diseases [11–13]. Testing for exogenous DNA (e.g., viral or bacterial) within an organism is another powerful target sequence detection application. Screening a population for Hepatitis C infection requires detecting only a short sequence (∼12 bp) within the 9.6 kb transcript [14]. The same concept applies to *in vivo* or *in situ* “bugs”, like Borrelia bacteria that causes Lyme disease [15], bacterial mouth flora that are biomarkers for cardiovascular disease [16], or food infected with *E. coli* or other bacterial pathogens.

Target sequence detection is typically performed by hybridization assays, such as microarrays [17], or by PCR-based techniques including primer extension PCR [18] and qPCR [19, 20]. Although these techniques are widespread, they require complex and costly device infrastructure to perform quantitation, using gel electrophoresis or some form of optics and fluorescence. By comparison, solid-state nanopores provide a nucleic acid sensor that is electronic, without the need for optics. Instead, the pore serially and electrically detects each DNA that passes through it, generating a distribution of 100-1000s of measurements within tens of minutes [21]. In this paper, we combine engineering of sequence-specific binding probes with the simplicity of nanopores to provide an electronic, single-molecule method for target sequence detection.

**Fig 1:**
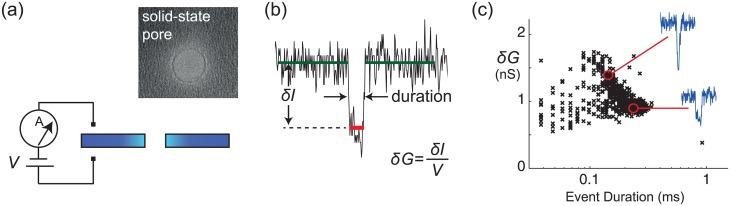
Single-molecule sensing with a nanopore device. (a) Schematic diagram of the setup with voltage V applied across a single nanopore fabricated in a solid-state substrate, while measuring the current through the pore. (b) A representative event caused by a 3.2 kb dsDNA passing through an 27 nm diameter nanopore at *V* = 100 mV (1M LiCl). Events are quantitated by shift conductance (*δG* = *δI*/*V*) and duration. (c) Scatter plot of *δG* versus duration for 713 events recorded over 10 minutes.

Unlike any other single-molecule sensor, the nanopore device can be packaged into a hand-held form factor at very low cost [22]. A solid-state nanopore is a nano-scale opening formed in a thin solid-state membrane that separates two aqueous volumes [23]. A voltage-clamp amplifier applies a voltage across the membrane while measuring the ionic current through the open pore (Fig 1a). When a single charged molecule such as a double-stranded DNA (dsDNA) is captured and driven through the pore by electrophoresis, the measured current shifts, and the shift depth (*δI*) and duration are used to characterize the event (Fig 1b). After recording many events during an experiment, distributions of the events are analyzed to characterize the corresponding molecule (Fig 1c).

While it is straightforward for a nanopore to detect dsDNA in a variety of lengths (50-50,000 bp) provided the measurement setup is sufficiently sensitive, the signal cannot infer the sequence of the DNA. Since our study is focused on detecting the presence of specific sequences within dsDNA, we design probes that bind to the target sequence of interest with high specificity, and such that the DNA/probe event signatures are sufficiently distinct from DNA alone. In this way, a population of DNA molecules could be screened to determine if a target sequence is present, by first incubating with the probes, and then measuring to see if the event population shift reveals the presence of the DNA/probe complex.

Nanopore detection of peptide nucleic acid (PNA) probes bound to specific sequences within dsDNA was performed first in the novel work by Meller and co-authors using precise and small (<5 nm) nanopore geometries [24, 25]. In an initial work, a bisPNA was used to bind two different 8 bp target sequences 855 bp apart within a 3.5 kb DNA [24]. Since bisPNA can only bind purines, the set of target sequences is limited to only homopurine or homopyrimidine stretches, with the latter achieved by binding the bisPNA to the complementary purine sequence. The work in [25] used the more versatile *γ*PNA. Since *γ*PNA creates a smaller feature than bisPNA, a smaller precision pore (3.7 nm) and asymmetric salt buffer (0.2M/1M KCl) were required for detection. By using precise pore geometries, these works permitted not just detection but “barcode” reading of PNA-bound sites. Other works have utilized PNAs for selective detection of single-stranded nucleic acids, with biological pores [26] and metallic (gold) nanopores [27]. We utilize both bisPNA and *γ*PNA and are the first to augment their utility for nanopore detection by chemically modifying their backbone to incorporate molecular handles. The handles in turn permit binding or covalent linking of other off-the-shelf molecules that increase the effective probe size, and thereby facilitate nanopore detection.

A key advantage of solid-state nanopore technology is that it can be made using scalable fabrication techniques at very low cost [28], and incorporated into small form factors [29] (Fig 1a). However, to be commercially viable the required fabrication tolerances cannot be too high, or the yield will be unacceptably low. While exploratory research studies often employ precise nanopore geometries [24, 25, 30–33], it is challenging to make two or more solid-state pores that have precisely the same geometry and electrical performance. In fact, two pores that visually appear the same via high resolution images can exhibit considerable variation in the dynamics of DNA passage [34, 35] and electrical noise [36]. Nanopores in ionic solutions are also often subject to size instability, growing in some instances significantly (>1nm) over the course of an experiment (>1h) and complicating analysis of biomolecular signatures which are highly dependent on the pore size. For these reasons, we favor an approach in which the reagents are constructed so that the detection problem is tolerant to a range of nanopore sizes and in-situ pore enlargement [37].

## Materials and Methods

### DNA binding fragments

The initial 324 bp DNA fragment for bisPNA invasion was synthesized (Life Technologies) to contain a single binding site (GGGAAAG) in the middle of the fragment. Additional material for experimentation was created using PCR amplification with forward/reverse primer binding sites included at the 5′ and 3′ ends of the original fragment. The 300 bp fragments containing a binding site for the *γ*PNA targeting the CFTRΔF508 mutation (AAATAAATACTTATAGCAAAAA) or the wild-type sequence (AAATAAATACTTTCTATAGCAA) were synthesized and later amplified in a similar manner.

### PNA molecules

The bisPNA (PNA Bio) has the following sequence:

$$\begin{array}{c} \text{KK-Cys-O}—\text{CTTTCCC}—\text{O-Cys-O}—\text{JJJTTTJ}—\text{O-Cys-KK} \end{array}$$

where K is a lysine residue, Cys is a cyteine residue, O is a polyethylene glycol unit, and J is pseudoisocytosine. The 3 cysteine residues provide a chemical handle for linking up to three cysteine-reactive molecules.

The 22 bp binding *γ*PNA (PNA Innovations) has the sequence

$$\begin{array}{c} \text{2Ac-K-AAATAAATACTTATAGCAAAAA-K-O-alkyne-DBCO} \end{array}$$

where 2Ac-K is an acetylated lysine, O is a polyethylene glycol linker, and DBCO is a dibenzocyclooctyne group that was used in a copper-free “click” chemistry reaction with azide-modified molecules.

### DNA/PNA and DNA/PNA-PEG complex formation

To create the bisPNA-bound 324 bp scaffold, the cysteine containing PNA was first capped with a 2-fold excess of MTSEA for 1 hr at room temperature (RT) in 10 mM sodium phosphate, pH 6.5. Next, purified 324 bp target DNA was incubated with a 30-fold excess of bisPNA to target DNA for 2 hours at 50°C in the same buffer. The DNA/bisPNA complex was then spun down through a 7 kDa MWCO separation column (Thermo Scientific) to rid the sample of excess bisPNA. Prior to conjugation with PEG, the bisPNA was reduced using TCEP (Thermo Scientific) for 15 min at 37°C. For labeling of the DNA/bisPNA complex with PEG, the sample was incubated with a 1000-fold excess of 5 or 10 kDa PEG-maleimide (Nanocs) for 3 hrs at RT. The DNA/bisPNA/PEG construct was once again cleaned up using the 7k MWCO sizing column prior to nanopore analysis. All molecules were stored at -20°C prior to use.

For detection of the CFTRΔF508 mutation, the 300bp DNA containing the mutation was incubated with a 300-fold excess of DBCO labeled *γ*PNA in a pH 7.0, 10 mM sodium phosphate buffer for 2 hrs at 60°C. This complex was then labeled with 100-fold excess of 5kDa azide-PEG (Nanocs) overnight at RT. The sample was cleaned up using a 7 kDa MWCO column before nanopore experimentation. All molecules were stored at -20°C prior to use.

### PAGE-EMSA

In order to analyze the purity of the DNA and resulting DNA/PNA or DNA/PNA/PEG complexes, Polyacrylamide Gel Electrophoresis Electromobility Shift Assays (PAGE-EMSA) was performed. PAGE-EMSA was completed using 5%, 10%, or 4-20% TBE gels in pH 8.3, TBE buffer for 20 minutes at 100 V followed by 0.5-2 hours at 150 V. The gels were stained with Sybr Green nucleic acid gel stain (Life Technologies), and visualized using a Bio-Rad Gel Doc EZ Imager or UV light.

### General nanopore methods

Experiments using low-stress silicon nitride membranes 10 and 30 nm thick (Norcada) had pores formed by one of two methods. TPG pores were formed in 30 nm membranes using a helium ion microscope (HIM) [38, 39] through a collaboration with Carl Zeiss Microscopy (details in SI). Pores at the University of Ottawa were formed in 10 nm membranes directly in a buffered solution using controlled dielectric breakdown [40–42]. Pores were enlarged using pulses of reduced electric field strength [37]. The chips for CFTRΔF508 detection used 20 nm membranes and TEM-formed nanopores adjacent to bottle structures and are described in [43], with the bottle on the side opposing the reagents and therefore not playing a role in capture or event signatures of reagents. Experiments were conducted at 23°C in 10 mM HEPES/KOH or 10 mM Tris/HCl with 1 mM EDTA, at pH 8, and 0.1 or 1 M LiCl or 1M KCl using custom flow cells. With the exception of the data in Fig 1, a commercially available voltage-clamp amplifier (AxoPatch 200B for data from uOttawa, MultiClamp 700B for data from TPG, both Molecular Devices, Sunnyvale, CA) was used to apply transmembrane voltage and measure ionic current, with the 4-pole Bessel filter set at the reported bandwidths. A digitizer (Digidata 1440A, Molecular Devices, or National Instruments USB-6351 DAQ card) stored data sampled at 250 kHz. Each reagent was added at the reported concentration into the voltage-negative chamber during nanopore experiments, and following two flushes of fresh buffer for cases in which reagents were already present in the chamber. A summary of all conditions and nanopores used is reported in the SI (S10 Section).

### Data processing

All numerical analysis and data processing was done using custom code written in Matlab (2014, The MathWorks) or Python. Events are flagged and extracted if any sample falls below 5 times the standard deviation (*σ*) of the open channel signal, with *σ* computed using the period between every pair of flagged events. Each extracted event contains all samples adjacent to the sample(s) below 5*σ* up to the first samples below 1*σ*. Events are rejected from analysis if: they do not return to within 1*σ* (e.g., by truncation during data recording); if the signal-to-noise ratio of the minimum sample divided by *σ* is less than 5; or if the duration exceeds 10 ms. Each open channel duration is the time between every pair of extracted events, and the durations are used to compute the capture rate by fitting an exponential distribution to the data (a least-squares fit, as detailed in [44]) with the *R*^2^ value reporting the goodness of the fit. The open channel conductance values are used to track the evolution of the nanopore size and evaporation (detailed in SI). For each event, the reported duration is the time-width at half maximum. Each event *δG* value is the mean of all samples below 1*σ* after trimming the number samples at the start and end of the event that correspond to the rise time *t*_*r*_, thereby removing the affects of the low-pass filter. The value for *t*_*r*_ is determined by the amplifier and bandwidth setting, as follows: Using the Axopatch 200B with the Bessel filter at 100 kHz and 10 kHz, the composite bandwidths are 55.6 kHz and 9.8 kHz, with 10-90 rise times (*t*_*r*_) of 6.1 us and 35.4 us, respectively (detailed in SI). The MultiClamp 700B has an effective bandwidth matching the 30 kHz Bessel filter bandwidth, with 10-90 rise times of 12 us. For events shorter than 2*t*_*r*_ in duration, *δG* reports the maximum shift (rationale in S1 Section). We used median and interquartile range (IQR) values to calculate the most likely event duration and the spread for each data set. The IQR is the range between the 75th percentile and the 25th percentile. Thus, the IQR includes about 50% of the data and is a measure of statistical dispersion that minimizes the effect of outliers.

## Results and Discussion

Our study is focused on using a nanopore to detect the presence of probes bound to specific sequences within dsDNA, and thereby signaling the presence of the target sequence. The ideal probe binds the target sequence with high affinity and selectivity, allowing the probe to remain bound during the course of the test, and without cross-reacting with nucleic acid stretches nearly identical to the target sequence. We use PNA molecules as the probe that binds to target sequences within dsDNA. PNA molecules contain a peptide backbone and nucleic acid bases, and invade dsDNA to base pair with their cognate sequence with high affinity [45–48]. We first present results using a bisPNA molecule to enable detection of a single 7 bp sequence centrally located within 324 bp dsDNA. Next, we show detection of a 22 bp sequence using a *γ*PNA as the probe, centrally located within a 300 bp DNA. Both bisPNA and *γ*PNAs are chemically modified to provide sites to which bulk-adding molecules can be incorporated, by binding or covalent linking. We show that these bulk-adding molecules are necessary to achieve target-sequence detection using a range of nanopore sizes and geometries. We also provide a mathematical framework for assessing the statistical significance of detection of an event subpopulation in a nanopore assay. The framework is simple to implement and accommodates a generic detection criteria that can be based on any chosen set of metrics used to quantitate events (i.e., not just *δG* and duration), while also accounting for false-positives.

### Chemically modified bisPNA probes

**Fig 2:**
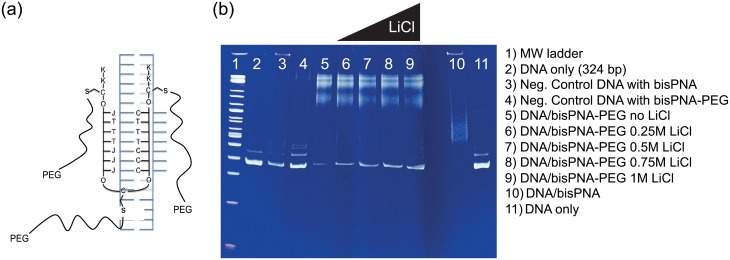
The bisPNA probe binds to dsDNA in conditions compatible with nanopore experiments, with and without a PEG payload. (a) The cysteine substituted bisPNA (black, U-shape) is bound to 324 bp scaffold dsDNA (blue) making a triplex helix. A modified cytosine is used that has less pH dependence when making Hoogsteen contacts (J). The two halves of the PNA are separated by a flexible PEG linker (O) that has a cysteine (C) amino acid in the middle. Lysines (K) are added at each end to increase the stability. PEGs containing maleimides react with the cysteine residues (C) in the PNA creating the DNA/bisPNA-PEG complex. (b) The DNA/bisPNA complex was found to be stable in up to 1M LiCl over a half-hour incubation at room temperature, indicating that the majority of the complex will be intact throughout the duration of the nanopore assay. Image shows 10% PAGE EMSA with lanes: 1) high molecular weight ladder, 2) DNA (324 bp), 3) DNA (negative control with scrambled 7 bp target sequence) with PNA, 4) DNA (negative control) with PNA-PEG (5 kDa), 5) DNA/PNA-PEG (5 kDa) and 10) DNA/PNA. Lanes (6-9) are DNA/PNA-PEG (5 kDa) after 30 min incubation in increasing LiCl concentrations (0.25, 0.5, 0.75, 1M).

A bisPNA molecule consists of two PNA halves that are separated by a flexible PEG linker, with the first half binding to the cognate sequence using Watson-Crick base pairing and the second half binding via Hoogsteen face pairing [46, 48] (Fig 2a). Each bisPNA molecule contained two terminal lysines at both ends to increase the affinity for the target site. We further modified the bisPNA to contain 3 cysteine residues to provide a chemical handle to bind up to three cysteine-reactive PEG molecules (i.e., the bulk-adding PEGs). Because PNAs have long been considered potential modulators of gene transcription, the conjugation of PEG to PNA has previously been explored to both increase solubility and enhance cellular uptake of the molecule [49]. This work utilized a conjugation chemistry that required complex and long (3 d) incubation conditions. By contrast, the thiol-maleimide chemistries utilized here are simple and proceed rapidly (hours). With the PEG-binding feature, we were able to compare the nanopore event signatures for DNA/bisPNA and DNA/bisPNA-PEG, while varying the PEG size (5, 10 kDa) and the nanopore size (6-50 nm diameter). The 5 kDa PEG is 36 nm in length (∼105 bp equivalent), while the 10 kDa PEG is 72 nm in length (∼210 bp equivalent). To ensure these complexes were correctly synthesized and amenable to nanopore detection, we first performed electrophoretic mobility shift assays (EMSAs) to test their biochemical quality, compare shift profile, and examine stability while varying salt concentration (Fig 2b).

Three well-defined bands are visible at the top of lanes containing DNA/PNA-PEG (5 kDa) complexes (Fig 2b, lanes 5-9). As observed by Hansen *et al*. [50], this is likely due to the three different complex types (complex I, II and III) formed by bisPNA associating with DNA. A separate EMSA with DNA/bisPNA-PEG and a similar bisPNA that contained only one reactive site for PEG also produced the three bands (not shown), suggesting the three full-complex bands are present regardless of PNA sequence and PEG number (up to 3). Given the stability of the DNA/bisPNA complexes with and without PEG observed for 30 minutes in 1M LiCl with EMSA, we performed nanopore experiments with these reagents using ∼30 minute recording periods. Recording periods longer than 30 minutes were sometimes used, in which cases the reagents produced consistent trends in capture rate and event property distributions for the entire period, suggesting that complex stability persisted beyond 30 minutes.

### Nanopore detection of bisPNA on 324 bp DNA

**Fig 3:**
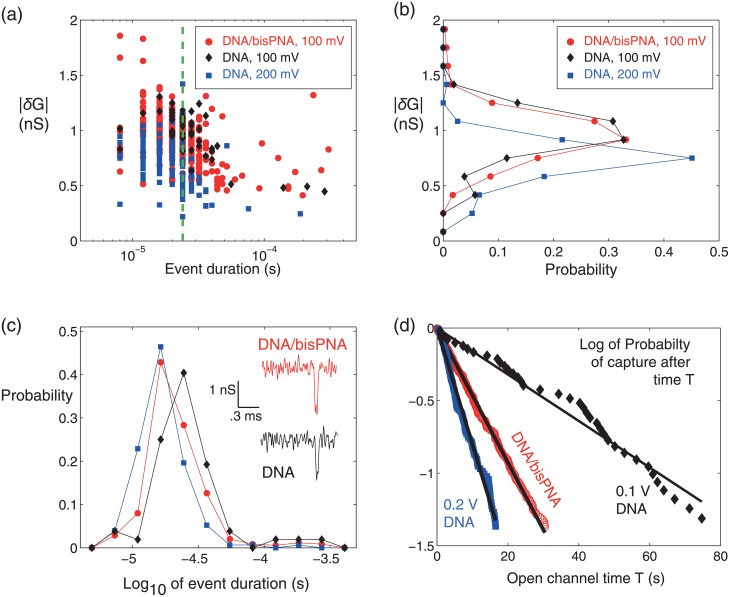
Short DNA and DNA/bisPNA events were indistinguishable in a ∼7 nm pore. (a) Population of *δG* vs. duration for all events in experiments with DNA alone (10 nM, 100 and 200 mV) and DNA/bisPNA (10 nM, 100 mV) show that most events are too fast (left of the 24 *μ*s resolution, green dashed line) to resolve full amplitude depth. (b) Event *δG* histogram for all events at least 24 *μ*s in duration shows that DNA and DNA/bisPNA are indistinguishable at 100 mV. (c) Duration histogram and representative events for DNA and DNA/bisPNA at 100 mV. (d) On the natural-log scale, the fraction of open channel times faster than time T for the three data sets are shown, with the data fit by a straight line (single exponential probability distribution, Methods).

We performed nanopore experiments to compare unbound DNA with bisPNA-bound DNA, to test whether the PNA-bound 7 bp target sequence could be detected. The DNA alone sample at 10 nM was run first, followed by a chamber flush and addition of 10 nM DNA/bisPNA using the same pore in 1M LiCl. Event distributions were recorded for the DNA and DNA/bisPNA experiments and plotted. Fig 3a shows the *δG* vs. duration event plot, with the corresponding event histograms for *δG* and duration in Fig 3b and 3c, respectively. Although a few events last long enough to hit full amplitude depth (Fig 3c), most events are faster than the time resolution of the instrument (24 *μ*s, Methods, dashed line Fig 3a). For plotting purposes, we quantitate event duration and report *δG* based on the max current shift value for all events shorter than 24 *μ*s in Fig 3a and 3b. Since the majority of events are too fast to resolve the true mean *δG*, the DNA and DNA/bisPNA populations cannot be discriminated by amplitude (Sec. 1 of SI).

A distinct change in capture rate was observed between the DNA and DNA/bisPNA complexes at the same concentration and voltage. A buffer only period was recorded first, producing only 2 events in 17 minutes that can be attributed to aperiodic noise (SI). Subsequently, the DNA produced 52 events in 49 minutes at 100 mV (0.016 1/sec, R^2^ = 0.974), and 153 events in 35 minutes at 200 mV (0.08 1/sec, R^2^ = 0.99). The decrease in *δG* values for DNA events at higher voltage further validates that the events are not hitting full amplitude depth (mean *δG* is 0.98 ± 0.2 nS at 100 mV, and 0.72 ± 0.18 nS at 200 mV). If events were hitting full depth, the *δG* histogram at 200 mV in Fig 3b would overlay or be higher than the 100 mV population [51]. The PNA-bound DNA had a higher capture rate than DNA at 100 mV, producing 350 events over 126 minutes. The PNA-DNA experiment was recorded as three 30 minute epochs of 10 nM complex at 100 mV, with each epoch separated by 2x flushing of the chamber (S2 Section). The capture rates for all three periods were conserved, with an all-event capture rate of 0.046 1/sec (R^2^ = 0.999). This increase in capture rate from DNA to DNA/bisPNA at 100 mV is an apparent indicator of probe-bound complex (Fig 3d), as observed in comparable studies [52, 53].

It is likely that many of the DNA and DNA/bisPNA molecules are passing through this nanopore undetected. One indicator is that most detected events are not hitting full depth, and are thus on the boundary of the resolution limit of our setup. Also, the capture rate of DNA (1 per minute) at 10 nM and 100 mV is much lower than for nanopores that are small enough to produce amplitude-resolvable signals [54]. The relative increase in capture rate for DNA/bisPNA suggests that a larger portion of these molecules is being detected than for DNA. A key component of the mathematical framework presented later in the paper is that it does not require all molecules passing through the pore to be detectable in order to discriminate bound vs. unbound DNA events; what is required is for bound-DNA events to have an event signature that is sufficiently different from unbound DNA events. This is also the case in other nanopore assays [52, 53]. Since the geometry of the pore used did not produce distinguishable event signatures for bisPNA bound vs. unbound DNA, we sought next to change the nanopore geometry.

Before comparing results with the same reagents and different nanopores, it is helpful to define a quantitative metric with which to compare the nanopores. To this end, we estimated the nanopore diameter from the time evolution of the open channel conductance, using the two models presented in [55] (SI). The modeled diameter range is 6.3-6.6 nm during the DNA alone experiment (S2 Section) and 6.6-7.5 nm during the DNA/bisPNA experiment (S2 Section). Thus, for a pore ∼7 nm in diameter in a 30 nm membrane, we discovered that we could not discriminate event signatures with and without the bisPNA bound to the DNA. Essentially, these complexes pass through the pore too fast to resolve the true *δG* value, and the pore was too large in diameter and length to produce a deep enough *δG* value above the measurement noise.

**Fig 4:**
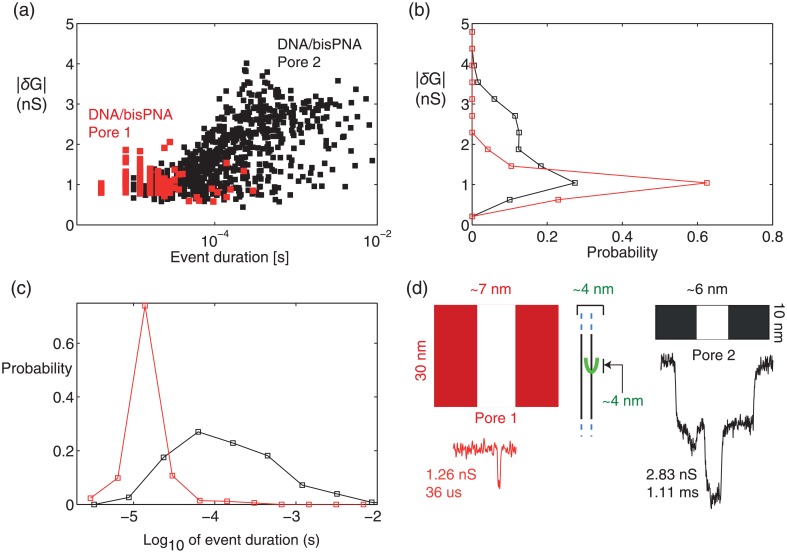
A single bisPNA on DNA is resolvable with a ∼6 nm pore. (a) Population of *δG* vs. duration for all events in the experiment with DNA/bisPNA (22 nM, 100 mV) with the ∼6 nm pore, overlaid with the DNA/bisPNA data from Fig 3. The event *δG* histogram (b) and duration histogram (c) shift significantly by using the smaller pore. (d) Schematics of the nanopores and the complex, all sized using a common scale for visual comparison, and a representative event from each experiment (reporting *δG*, duration). The bisPNA creates a bulge on DNA approximately ∼4 nm in width and length.

The same DNA/bisPNA complex was tested using a smaller pore, reducing the membrane thickness from 30 nm to 10 nm, and reducing the diameter from ∼7 nm to ∼6 nm (S2 Section). With this smaller pore formed *in situ* by controlled dielectric breakdown [40, 41] (Methods), deeper and longer lasting events were present, producing signatures clearly distinguishable from the DNA alone and DNA/bisPNA events produced with the larger pore. Fig 4a–4c shows event populations for the DNA/bisPNA reagent (22 nM, 1M LiCl) at 200 mV using the smaller pore, with the events from the larger pore experiment (DNA/bisPNA, 100 mV, Fig 3) overlaid. In terms of summary statistics, the larger pore produced a faster duration (median = 20 *μ*sec, IQR = 12 *μ*sec) and shallower mean *δG* (0.95 ± 0.2 nS) than for the smaller pore (median = 118 *μ*sec, IQR = 316 *μ*sec, mean *δG* = 1.71 ± 0.8 nS). A representative event signature attributable to DNA/bisPNA with the smaller pore is shown in Fig 4d, along with the sizes of the two pores and the complex all compared in a common scale. In terms of volume occlusion, one can approximate the pore and DNA (2.2 nm) as cylinders, with the PNA creating a bulge approximately 4.4 nm in diameter and 4.3 nm in length. The 4.3 nm length estimate includes the 7 bp binding footprint and the triethylene glycol linker and three amino acids. With the bulge located fully in the pore, the DNA/bisPNA occludes only 4% more than DNA alone in the larger pore, but 17% more in the smaller pore, indicators that are consistent with only the smaller pore providing the resolvable difference in event signature. A variety of event signature patterns attributable to DNA/bisPNA complexes with the smaller pore were recorded (S3 Section). The experiment also generated significantly more events (767, over 23 minutes, 0.56 1/sec), a byproduct of using a higher concentration (22 nM vs. 10 nM) and a higher electric field strength, from 3.33 mV/nm increased 6-fold to 20 mV/nm. The primary difference between the pores is the apparent reduction in the speed of the complex through the smaller pore, a trend consistent with other studies in which the nanopore size approaches the size of the largest feature that passes through the pore. In general, a drawback of making pores closer to the size of the largest feature is the increased risk in pore clogging.

In summary, our results suggest that a ∼7 nm diameter pore in a 30 nm membrane cannot resolve the ∼4 nm bulge created by a single bisPNA bound to dsDNA, whereas a ∼6 nm pore in a 10 nm membrane can resolve the PNA. This is not too surprising, since a comparable bisPNA (8 bp instead of 7 bp footprint) bound to DNA was detected using a smaller pore still (<5 nm in diameter) in a 30 nm membrane [24]. We sought next to test PNA probes with increased size, with the aim of enabling PNA-bound target sequence detection using larger nanopores.

### Nanopore detection of PEG-bound bisPNA on 324 bp DNA

**Fig 5:**
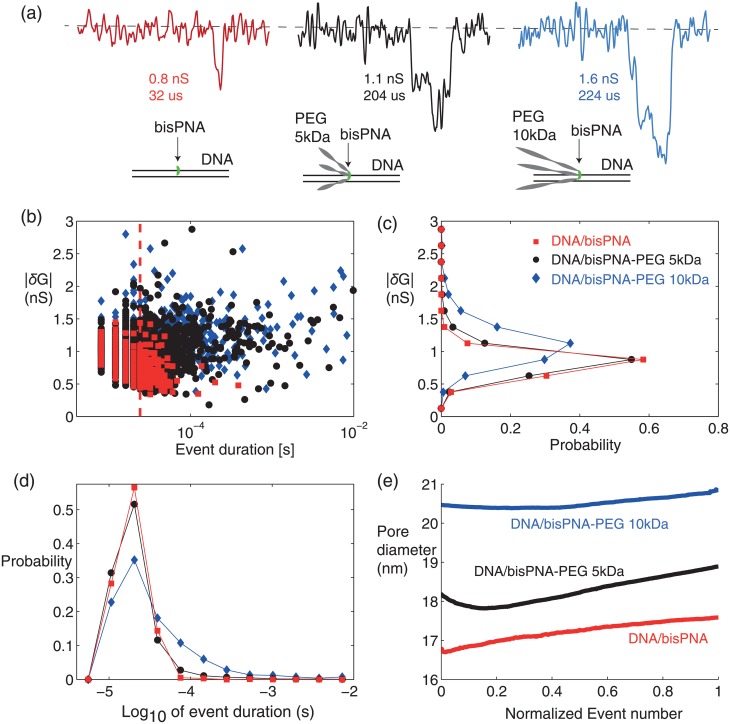
A single bisPNA on DNA is resolvable with a 17-21 nm pore by adding bulk through bisPEG-PNA linking. (a) Representative events: DNA/bisPNA (left), and DNA/bisPNA-PEG with up to 3 PEGs on each PNA, and PEG sized 5 kDa (middle) and 10 kDa (right). Molecule depictions show linear PEG and DNA sized to scale for visual comparison. (b) Population of *δG* vs. duration for all events in each data set, each at 2 nM and 100 mV in 1 M LiCl, with *δG* histogram (c) and duration histogram (d) shifted by adding PEGs of increasing size. (e) Evolution of the modeled nanopore diameter, using the time history of the open channel conductance (S2 Section), spanning 30 min (DNA/bisPNA), 58 minutes (DNA/bisPNA-PEG 5 kDa) and 40 minutes (DNA/bisPNA-PEG 10 kDa).

Prior to testing DNA/bisPNA-PEG complexes, a 5 kDa PEG alone control at 2 nM was tested in 1M LiCl at 100 mV, producing only 13 events in 30 minutes that could not be differentiated from aperiodic noise spikes (SI). This result and the neutral charge of the PEG suggest that any background PEG will not produce an appreciable number of events. Both PEG alone and bisPNA-PEG negative controls produced almost no events with other pore sizes used also (SI). After flushing PEG from the chamber, 2 nM DNA/bisPNA was added, producing 991 events over 30 minutes (0.625 1/sec, R^2^ = 0.999). As in the DNA/bisPNA experiment with the ∼7 nm pore (Fig 3), the larger ∼17 nm pore produced events with the majority (76.9%) too fast to resolve their true amplitude (Fig 5, S4 Section). The events produced a median duration of 20 *μ*sec (IQR = 12 *μ*sec) and a mean *δG* of 0.81 ± 0.16 nS. DNA alone experiments for this and for larger pores also produced events too fast to resolve their true amplitude, and with a lower event rate than for DNA/bisPNA.

We next tested DNA/bisPNA-PEG (5 kDa) using the same nanopore at 100 mV. The complex was tested at 2nM in three epochs (32, 31 and 58 minutes), with 2x chamber perfusion prior to each epoch and the nanopore now 18-19 nm in diameter (Fig 5e, S4 Section). All three epochs produced consistent event amplitude and duration distributions and capture rates: the first epoch produced 2957 events (1.567 1/sec, R^2^ = 0.999); the second produced 2887 events (1.58 1/sec, R^2^ = 0.999); the third produced 4218 events (1.22 1/sec, R^2^ = 0.999) with event distributions for the third epoch shown in Fig 5. Visually, the DNA/bisPNA-PEG complexes produced an increase in the number of deeper and longer lasting events than were present with DNA/bisPNA complexes without PEG (Fig 5a and 5b, S4 Section). The histograms (Fig 5c and 5d) and summary statistics show that the DNA/bisPNA-PEG events produced a median duration comparable to DNA/bisPNA (16 *μ*sec, IQR = 12 *μ*sec), with a modest increase in the *δG* mean and variance (0.86 ± 0.21 nS).

The same pore was next used to test the larger DNA/bisPNA-PEG (10 kDa) complexes. As before, after perfusion and a period of recording buffer only, the DNA/bisPNA-PEG (10 kDa) was tested at 2nM in three consecutive 40 minute epochs at 100 mV, each separated by 2x chamber perfusions. The nanopore enlarged to 20-21 nm in diameter (Fig 5e) by the action of voltage applied for a prolonged period (SI). The third epoch produced 818 events (0.422 1/sec, R^2^ = 0.998) with event distributions shown in Fig 5. The DNA/bisPNA-PEG (10 kDa) complexes produced a more pronounced increase in deeper and longer lasting events, compared to DNA/bisPNA complexes without and with 5 kDa PEG (Fig 5, S4 Section). In terms of summary statistics, the duration (median = 24 *μ*sec, IQR = 36 *μ*sec) and mean *δG* (1.11 ± 0.3 nS) increased. If each PEG were linearized and laying flat against the DNA, the 5 kDa PEGs (∼105 bp length) would not extend beyond the length of the DNA (162 bp half-length), while the 10 kDa PEGs (∼210 bp length) would extend beyond the DNA, a configuration that could produce the observed increase in event durations. It is plausible that the PEG transiently and stochastically interacts with the pore wall during translocation, which is more likely to occur and for longer with the larger PEG size. The noise performance of the nanopore was comparable for all three data sets shown (S4 Section).

Since the bisPNA can form complex I, II or III structures on DNA [50], and have 0, 1, 2 or 3 PEGs linked, we expected and indeed observed heterogeneity in the event signatures observed for the two PEG sizes used, still with an overall trend of an increase in the number of deeper and longer lasting events as the PEG size increased (S4 Section). The DNA/bisPNA-PEG (10 kDa) complexes were also tested in larger nanopores in the same 30 nm membranes, estimated to be 36 nm and 50 nm in diameter. As with the ∼20 nm pore results, the full complex alone produced deeper and longer lasting event signatures in these larger nanopores, when compared to negative controls (DNA alone, bisPNA-PEG and/or 10 kDa PEG alone) that gave either faster and shallower events or no events (S5 and S6 Sections). While PEG-bound DNA/PNA signatures are visually apparent, a more quantitative metric is desired to permit assigning confidence to each cumulative detection result. The mathematical criterion presented in the next section provides this capability.

### Mathematical framework for assigning statistical significance to detection

In general terms, there are two categories of molecules in the chamber above the pore: type 1 are all the background molecules, and type 2 are the molecules of interest. For our application, type 1 molecules are unbound DNA, free PNA, free PEG and PNA-PEG molecules, while type 2 molecules are DNA/PNA-PEG complexes. It must be experimentally established that the chosen bulk-adding molecules (i.e., PEG) do not bind nonspecifically to DNA and thereby undermine our ability to discriminate type 1 and 2 molecules. The goal is to detect the presence of type 2 molecules in bulk solution, and to assign statistical significance to detection.

The first component of the framework is to identify an event signature that is almost absent in type 1 events but is present in a significant fraction of type 2 events. An event is then “tagged” as being type 2 if the signature criteria is met for that event. A signature could depend on *δG*, duration, the number and characteristics of levels within each event [56], and/or any other numeric values computed from the event signal. For a given assay, the tagging signature is initially established using data from control experiments both with and without type 2 molecules present. For our target sequence detection assay (Fig 5), the data suggests that a minimum duration threshold is a viable signature. For target sequence detection assays in which the presence of type 2 molecules from a sample is unknown, the duration threshold value would be established before testing the sample by first running the negative control on the same nanopore. The negative control would be a DNA with the same length as the target-containing DNA, and without the target sequence, perhaps also with an equivalent amount of PNA-PEG probes to be used during the incubation reaction between the sample and probes. The mathematical details below restate these procedures in an algorithmic format, assuming the tagging signature has already been identified.

Defining *p* as the probability that a capture event is type 2, we can approximate the probability that a capture event is tagged using the quantity

$$\begin{array}{c} Q\left(p\right)=\frac{\text{Number}\;\text{of}\;\text{tagged}\;\text{events}}{\text{Total}\;\text{number}\;\text{of}\;\text{events}}=\frac{1}{N}\sum_{j=1}^{N}X_{j} \end{array}$$

where *N* is total number of all events. Each *X*_*j*_ is a sample value of the random variable *X*, with *X* defined to be 1 or 0 when an event is tagged or untagged, respectively, and having a Bernoulli distribution. Observe that *Q*(*p*) is a sample mean of *X* that can be readily computed and utilized as follows:

- In a control experiment or set of experiments without type 2 molecules (*p* = 0), the false-positive value *Q*(0) is determined with good accuracy from a large number of capture events;
- In a detection experiment for which the presence of type 2 molecules in bulk solution is to be determined, the value for *p* is unknown, and *Q*(*p*) is computed;
- Based on a confidence interval *Q*(*p*) ± Q_*_ generated for *Q*(*p*), we test if
  $$\begin{array}{c} Q\left(p\right)-Q_{*}>Q\left(0\right) \end{array}$$ (1)
  If Eq (1) holds true, we can say that *Q*(*p*) > *Q*(0) is statistically sound and thus *p* > 0 is statistically sound.

The 99% confidence interval can be computed as $Q_{*}=2.58\sqrt{Q(p)(1-Q(p))/N}$, which is derived in the Supporting Information document along with alternative methods and the option to vary the confidence level (e.g., 90 or 95% confidence). If Eq (1) does not hold true, we cannot assign statistical confidence to the result. In the context of the data in this paper, to state that *p* > 0 is statistically sound is to be able to assign statistical confidence to target sequence detection. Of course, the same test can be used for any other assay in which a distinctive nanopore event criteria can be derived with which to tag any target molecule of interest.

Since no two nanopores are the same, it is sensical to establish *Q*(0) first with a given nanopore by running a negative control first prior to testing for the presence of the desired complex. Depending on the assay, and other prior negative controls, it may not be necessary to run all negative controls for each new nanopore. For example, PEG alone produced only rare events that could not be distinguished from the rare aperiodic noise commonly observed in our experiments (SI) and by others [53, 57]. Thus, PEG alone is a negative control that is likely unnecessary for a new pore, provided other elements of the experiment remain at or near the values used previously. We next apply the method above to our data sets.

We now consider DNA/bisPNA-PEG complexes as the molecules of interest (“type 2”) that signal the presence of a target sequence, utilizing the data shown in Fig 5. The nanopore initially tested PEG alone and DNA/bisPNA prior to measuring DNA/bisPNA-PEG. An example signature for tagging an event as type 2 is if the duration is longer than 50 *μ*sec. Using the DNA/bisPNA set as the negative control, the false-positive probability is *Q*(0) = 1.21%. From the DNA/bisPNA-PEG (5 kDa) data we have *Q*(*p*) ± Q_*_ = 6.09 ± 0.95%. Since *Q*(*p*) − Q_*_ = 5.14 > 1.21%, positive detection of DNA/bisPNA-PEG (5 kDa) is achieved with 99% confidence. Observe that detection with 99% confidence was achieved despite the similarity in the histograms (Fig 5) and indistinguishable aggregate statistics between the DNA/bisPNA and DNA/bisPNA-PEG (5 kDa) populations.

By applying the framework to the DNA/bisPNA-PEG (10 kDa) data, we see that the increased PEG size increases the margin for positive detection. For the DNA/bisPNA-PEG (10 kDa) data generated with the same pore (Fig 5), *Q*(*p*) ± Q_*_ = 25.9 ± 3.95% when tagging events as type 2 if the duration is longer than 50 *μ*sec. By using the DNA/bis-PNA data again to establish the false positive value, Eq (1) becomes *Q*(*p*) − Q_*_ = 21.97 > 1.21%, a much higher margin than for DNA/bisPNA-PEG (5 kDa). Note that the uncertainty margin (Q_*_) is larger for DNA/bisPNA-PEG (10 kDa) since DNA/bisPNA-PEG (5 kDa) had 5 times more events and Q_*_ ∝ 1/*N*.

A plot of *Q*(*p*) ± Q_*_ as a function of recorded event number *N* (S7 Section) shows the evolution of *Q*(*p*) and the attenuation of the error bars (±Q_*_) over time for the type 2 molecules considered (DNA/bisPNA-PEG 5, 10 kDa) compared to the false-positive threshold (*Q*(0) = 1.21%). The first-time that the lower error bar *Q*(*p*) − Q_*_ exceeds the false-positive line *Q*(0), and remains above this line, provides a time-to-results (TTR) value. The TTR estimates are 309 seconds and 163 seconds for the 5 kDa and 10 kDa PEG-bound DNA/bisPNAs, respectively, with the faster result for the larger and more easily detectable complexes. TTR is a value commonly used to assess molecular diagnostic assay performance. An analytic expression to model the TTR is provided in the SI.

As presented, the application of the framework requires manually choosing an event criteria that is the basis for tagging events. One can envision a means of automating the selection of such a criteria (e.g., using optimization [58]). However chosen, it is important to assess the robustness of the result by examining how tolerant the result is to changes in the criteria threshold value(s). For example, are DNA/bisPNA-PEG (5,10 kDa) molecules detected with 99% confidence if we change the duration threshold from 50 *μ*sec to 10, 100 or 1000 *μ*sec? Once each recording is finished, the final separation of the lower error bar *Q*(*p*) − Q_*_ above the false-positive line *Q*(0) is an indicator of result robustness. Additionally, a quantitative test of robustness is to compute the range of criteria threshold value(s) that preserve the 99%-confidence detection result. Using the data from Fig 5, a plot comparing *Q*(*p*) − Q_*_ for DNA/bisPNA-PEG (5,10 kDa) and *Q*(0) for DNA/bisPNA was generated while varying the duration threshold used to tag events as type 2 (S7 Section). The trends show that 99% detection confidence in preserved for any duration threshold in the ranges [28, 2900]*μ*s for DNA/bisPNA-PEG (5 kDa) and [12, 4600]*μ*s for DNA/bisPNA-PEG (10 kDa). Just as the (*Q*(*p*) − Q_*_) value can be computed and updated in real-time as the number of events *N* increases, the robustness margin (as shown in S7 Section) can also be computed and monitored in real-time.

The framework above also allows us to consider the viability of a multiplexed target sequence detection method. Specifically, consider a scenario in which the bisPNA-PEG (5 kDa) is used first as a probe for one target sequence of interest, and the bisPNA-PEG (10 kDa) is subsequently used as a probe for a second target sequence of interest. The question is whether both could be detected (sequentially) with confidence. First, we already established that DNA/bisPNA-PEG (5 kDa) was detected with 99% confidence above background. Subsequently, due to the large fraction of DNA/bisPNA-PEG (10 kDa) events exceeding 50 *μ*sec, the margin in Eq (1) is still maintained when DNA/bisPNA-PEG (5 kDa) are also considered type 1 molecules. Specifically, *Q*(*p*) − Q_*_ = 21.97 > *Q*(0) = 6.09%. Thus, the framework applied to these data establishes that DNA/bisPNA-PEG (5 kDa) and subsequently DNA/bisPNA-PEG (10 kDa) molecules are present with 99% confidence. A sequential multi-target detection assay such as this would be valuable when two or more genetic markers are needed to obtain a more informed result. An example would be to test first for the pathogenic bacteria Staphylococcus aureus by targeting a unique sequence within the nuc gene, and subsequently test if it harbors antibiotic resistance by targeting the mecA gene (Methicillin-resistant S. aureus, MRSA). To be more broadly applicable, the remainder of the paper considers *γ*PNA instead of bisPNA as the target-binding probe.

### Nanopore detection of CFTRΔF508 gene mutation using PEG-bound *γ*PNA

This section presents the use of the more versatile *γ*PNAs that can bind to a larger set of sequences than is possible with bisPNA, as described in the Introduction. A single *γ*PNA probe was used to bind to a 22 bp target sequence within 300 bp DNA. The target sequence encompasses the CFTRΔF508 mutation, a one codon deletion in the cystic fibrosis transmembrane conductance regulator (CFTR) gene that has a significant positive correlation with cystic fibrosis incidence levels [59]. Since the sequence is comprised of pyrimidines and purines, *γ*PNA can be used but bisPNA cannot be used for detection. In this application, only one 5 kDa PEG payload was linked to the *γ*PNA using copper-free “click” chemistry [60]. Additionally, experiments were performed in low salt (100 mM LiCl) to increase DNA/*γ*PNA complex stability (SI).

**Fig 6:**
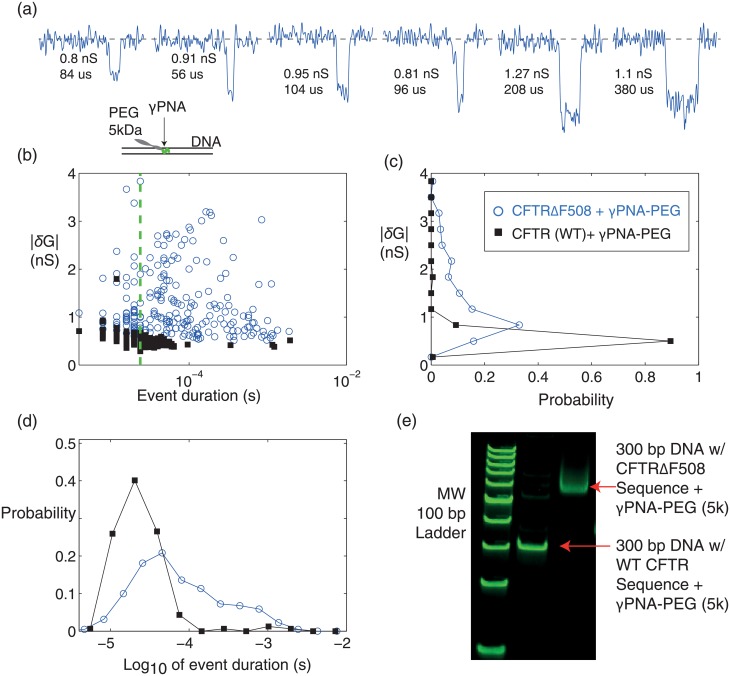
The 300 bp DNA/*γ*PNA-PEG 5 kDa complex is resolvable with a 26-36 nm diameter pore in 100 mM LiCl, providing positive detection of the CFTRΔF508 gene mutation. (a) Representative events with the pore initially at 26 nm in diameter, reporting *δG* and duration values. (b) Population of *δG* vs. duration for all 221 events over 52 minutes at 1 nM complex and 200 mV. Events span three pore sizes (26 nm, 32nm, 36 nm) that were enlarged by dielectric breakdown. The green line (24 *μ*sec) is the minimum duration for resolving *δG*. (c) *δG* histogram and (d) duration histogram of all events. (e) 5% PAGE EMSA shows the 22 bp *γ*PNA-PEG (5k) bound to the 300 bp DNA at the 22 bp target sequence that encompasses the CFTRΔF508 mutation (right lane). The *γ*PNA-PEG (5k) does not bind to the 300 bp DNA that has the wild-type (i.e., non-mutant) sequence (middle lane), showing target specificity. The sizing ladder is low molecular weight (left lane).

Nanopores were formed using TEM in 20 nm membranes, as described in [43] (SI). The DNA/*γ*PNA-PEG complex (1 nM, 200 mV) produced 221 events over 52 minutes. Detected events were exclusively attenuations, and not enhancements, consistent with other studies in 0.1 M LiCl at 200 mV [61]. Representative DNA/*γ*PNA-PEG events produced using a 26 nm pore (46 events, 18 min) are shown in Fig 6a. The pore was enlarged to 32 nm (30 events, 8 min) and then 36 nm (145 events, 26 min) using dielectric breakdown. The events for all three epochs were combined in the distribution plots (Fig 6b–6d) due to the lower capture rate at lower salt and since the event and noise characteristics remained consistent (S9 Section). As one measure of consistency, the three epochs produced 41%, 33% and 34% of events longer than 100 *μ*sec. In terms of summary statistics, the duration (median = 56 *μ*sec, IQR = 146 *μ*sec) and mean *δG* (1.28 ± 0.7 nS) were larger and more disperse that for DNA alone. A direct comparison between DNA alone (20 nm pore, 1 nM, 200 mV, 100 mM LiCl) and DNA/*γ*PNA-PEG events (S9 Section) shows a clear difference in nanopore signature when the *γ*PNA-PEG probe is bound, signaling the presence of the CFTRΔF508 mutation.

To further validate the assay, a separate nanopore experiment with the *γ*PNA-PEG and 300 bp DNA with the wild-type CFTR (i.e., non-mutant) sequence was tested (1 nM DNA, 0.1M LiCl, 200 mV, S9 Section). The wild-type CFTR + *γ*PNA-PEG events (162) were detected over 22 min with a 37 nm pore, and are overlaid on the CFTRΔF508/*γ*PNA-PEG events (Fig 6b–6d). In terms of summary statistics, the duration (median = 24 *μ*sec, IQR = 24 *μ*sec) and mean *δG* (0.55 ± 0.14 nS) were consistent with a 300 bp DNA alone control run just prior on the same pore (72 events, median = 22 *μ*sec, IQR = 20 *μ*sec, mean *δG* = 0.53 ± 0.1 nS). Following the WT CFTR and DNA alone assays, a *γ*PNA-PEG only control was also run producing only 5 events in 22 minutes. Finally, an EMSA assay equivalently showed the specificity of the probe for its target (Fig 6e).

The mathematical framework can be used to assign confidence to the result for detecting the CFTRΔF508 mutation. By tagging events that exceed 100 *μ*sec in duration, only 2.5% are tagged with the WT sequence and 35.3 ± 8.3% are tagged with the mutant sequence, a margin large enough for positive detection with 99% confidence (TTR = 4 minutes). Other criteria based on *δG* yield the same result. For example, by tagging events with *δG* > 1 nS, only 0.62% are tagged with the WT sequence and 50.7 ± 8.7% are tagged with the mutant sequence.

Given the ability of a nanopore to detect the CFTR sequence in a short DNA stretch, a diagnostic test for the ΔF508 mutation would be relatively simple. First, a small sample (e.g., cheek swab) is taken and an amplicon a few hundred base pairs in length is generated with PCR by using CFTR sequence specific primers that flank the mutation. The product is then incubated with a PNA-PEG, with the PNA matching the target sequence, and detected using a nanopore to reveal the presence or absence of the genetic mutation. A mutant-negative control can be run first to establish the false-positive % for the criteria chosen. The value of detecting probe-bound DNA, as opposed to just the DNA (PCR product) directly, is that it signals the presence of the target sequence with the specificity that is governed by the probe-DNA binding kinetics. This makes the quantitation step tolerant to the non-specific DNA amplicons that are commonly found in PCR reactions. The more common quantitation method of qPCR cannot decipher contaminants from targets. Moreover, while commercially available EMSA assays and optics-based assays are confined to lab settings and are time expensive, nanopores can give an answer in minutes and are amenable to scalable fabrication methods that can be integrated into portable formats [28, 62], which in turn broadens the prospective venues for target sequence detection.

Collectively, our results show single-target detection for short DNA (300 bp, 324 bp) that does not fold when passing through the nanopore. Applications that require a PCR amplification step prior to nanopore sensing will typically generate DNA lengths that are also too short to fold (<1 kb), making our results directly applicable to such cases. For applications with longer DNA (>1 kb), one must test if folding of the DNA undermines detection of a target-bound probe, with consideration that the number of possible folds increases with nanopore size and DNA length [63]. In initial tests, we demonstrated detection of multiple copies of a target 12 bp sequence within a 5.6 kb dsDNA using size-enhanced *γ*PNA probes (S8 Section), despite unbound DNA producing folded and unfolded event signatures. Future work will test if detection can be achieved with single-copy targets within long DNA.

## Conclusions

To improve the sensitivity of nanopores, researchers typically optimize the nanopore materials and geometry to detect DNA and features of interest on the DNA. In the context of target sequence detection, we take a PNA-based chemical biology approach that makes the sensing problem easier by making detection possible with a wide range of pore sizes. Simple and inexpensive circuity can be used to form and increase the size of pores *in situ* [42]. Regardless of how the pore is formed, voltage-pulse conditioning that increases pore size is often required to get sufficiently stable baseline performance before reagents can be tested [37]. This is not an issue with our target sequence detection assay since it does not require sub-nanometer precision pore geometries and can work for larger pores. As a corollary of our work, if the target sequence of interest is comprised of purely homopurines or homopyrimidines, bisPNA is a superior target-binding probe compared to *γ*PNA. This is because bisPNA has an additional DNA binding mechanism of Hoogstein face pairing that forms additional bonds between the two molecules. These additional contacts increase complex stability such that it remains bound in higher salt recording conditions, which in turn results in higher DNA capture rates and a faster time to results.

A nanopore-based technology that can detect the presence or absence of a target sequence from a DNA sample would have a variety of uses, particularly if the implementation is comprised of inexpensive disposable components with a reusable and inexpensive interface. In the context of cancer treatment, such a device could efficiently detect specific genetic mutations that are known to respond well to particular therapies (e.g., KRAS, HER2, EGFR, etc.), resulting in improved drug efficacy and an optimized-for-the-individual approach to treatment [64]. Alternatively, a PNA probe could bind to a genomic fragment to identify it, and the pore could test for methylation of CpG islands within promoter or repressor regions adjacent to the probe by a known distance, with methylation being tested by the presence or absence of zinc finger proteins. This would advance the work in [31] by providing the sequence context that is needed to inform the relevance of the detected methylation sites.

## Supporting Information

S1 Section Temporal resolution of nanopore measurements. Subsections cover computing the temporal response and event quantitation.
(PDF)

S2 Section Conductance models for estimating nanopore diameter. Subsections include comparing modeled diameters in a DNA and DNA/bisPNA experiment, and comparing modeled diameters in two DNA/bisPNA experiments.
(PDF)

S3 Section Representative events from DNA/bisPNA data with 6nm pore. (PDF)

S4 Section Comparing DNA/bisPNA and DNA/bisPNA-PEG with 17-21 nm pore. Representative events from DNA/bisPNA and DNA/bisPNA-PEG data sets are compared.
(PDF)

S5 Section Larger pore (36 nm) experiment with DNA/bisPNA-PEG 10 kDa. (PDF)

S6 Section Largest pore (50 nm) experiment with DNA/bisPNA-PEG 10 kDa. (PDF)

S7 Section Mathematical framework for assigning statistical significance to nanopore event subpopulation detection. The subsections present the framework in detail. A final subsection details application of the framework to data in Fig 5.
(PDF)

S8 Section Detection of 12 bp targets within 5.6 kb dsDNA using size-enhanced *γ*PNA probes. (PDF)

S9 Section Details on CFTRΔF508 detection on 300 bp DNA. (PDF)

S10 Section Summary of Experimental Conditions. Subsections include details on the Helium Ion Microscope methods for nanopore creation, and a Table that summarizes all experiment conditions.
(PDF)
