# Supplementary material for: Nanopore-Based Target Sequence Detection
Source: PLoS One. 2016 May 5;11(5):e0154426. doi: 10.1371/journal.pone.0154426 (PMC4858282; doi:10.1371/journal.pone.0154426)
Supplement: S1 Section — Subsections cover computing the temporal response and event quantitation. (PDF) [file pone.0154426.s001.pdf]

Supporting Information Sections for
Morin, *et al.*, "Nanopore-based target sequence detection"

S1. Temporal resolution of nanopore measurements

Computing the temporal response

The Axopatch 200B has a current-to-voltage converter response that corresponds to a single pole at 70 kHz ($\tau = 2.3$ us), which is then convoluted with a 4-pole low pass Bessel filter to produce the corresponding composite bandwidth and step response characteristics [1]. With the Bessel filter at 100 kHz and 10 kHz, the composite bandwidths are 55.6 kHz and 9.8 kHz, with 10-90 rise times (t_r) of 6.1 us and 35.4 us, respectively. The rise time is simply computed by simulating the step response of the composite transfer function and measuring the rise time, and the composite bandwidths are computed as the frequency at which the magnitude of the composite transfer function crosses -3 dB in the frequency domain (Bode plot). Thus, the converter has an appreciable affect on the measurement response at a filter bandwidth of 100 kHz bandwidth, but not at 10 kHz. The MultiClamp 700B has a current-to-voltage converter response that is faster than the 200B, with the effective bandwidth matching the maximum 30 kHz Bessel filter bandwidth, with 10-90 rise times of 12 us.

Event quantitation

The rise time t_r of the measurement at each bandwidth determines the detection limit of our event quantitation algorithms. As stated in the methods section, duration is computed as the width at half-maximum, which faithfully reports unfiltered pulse widths even when the pulse is faster than t_r [2]. Since events faster than $2t_r$ do not hit full amplitude depth, these events make amplitude-based comparative analysis difficult. Several papers have proposed methods to extract the unfiltered pulse shape, including duration and amplitude depth, for events faster than $2t_r$ [3, 4, 5]. These methods apply heuristics, by assuming a signal shape (or equivalently, basis functions) to represent the ideal signal if it were unfiltered [3, 4], or by assuming that without the filter the signal would be a pure pulse [5]. For the purposes of this work, no assumptions about the underlying signal shape are necessary in order to determine the statistical significance of our results, and so we take a simplistic approach and report the maximal value for δG for events shorter than $2t_r$.

References

[1] D R Garalde, C R O'Donnell, R D Maitra, D M Wiberg, Gang Wang, and W B Dunbar. Modeling the Biological Nanopore Instrument for Biomolecular State Estimation. *Control Systems Technology, IEEE Transactions on*, 21(6):2038–2051, 2013.

[2] Bert Sakmann, Erwin Neher. *Single-channel recording.* Springer, 2nd edition, 2009.

[3] Arvind Balijepalli, Jessica Ettedgui, Andrew T Cornio, Joseph W F Robertson, Kin P Cheung, John J Kasianowicz, and Canute Vaz. Quantifying Short-Lived Events in Multistate Ionic Current Measurements. *ACS Nano*, 8(2):1547–1553, February 2014.

[4] Z Gu, Y L Ying, C Cao, P He, and Y T Long. The Accurate Data Process for Nanopore Analysis. *Analytical Chemistry*, 2014.

[5] Daniel Pedone, Matthias Firnkes, and Ulrich Rant. Data analysis of translocation events in nanopore experiments. *Analytical Chemistry*, 81(23):9689–9694, December 2009.
