# Supplementary material for: Nanopore-Based Target Sequence Detection
Source: PLoS One. 2016 May 5;11(5):e0154426. doi: 10.1371/journal.pone.0154426 (PMC4858282; doi:10.1371/journal.pone.0154426)
Supplement: S2 Section — Subsections include comparing modeled diameters in a DNA and DNA/bisPNA experiment, and comparing modeled diameters in two DNA/bisPNA experiments. (PDF) [file pone.0154426.s002.pdf]

Supporting Information Sections for
Morin, *et al.*, “Nanopore-based target sequence detection”

S2. Conductance models for estimating nanopore diameter

We do not image nanopores to quantitate their diameter, but instead estimate the diameter using models of the open channel conductance, measured as $G = I/V$ with current I and voltage V. Models of conductance from the literature can be used to predict the diameter of the nanopore from the evolution of the open channel current over the course of the experiment. Specifically, G is computed between every pair of events as the average current divided by the voltage. We reference two models presented in [1].

The first model for G ignores any access resistance contributions to the total resistance (total resistance is the inverse of the total conductance), and depends on nanopore diameter d and membrane thickness L as:

$$G_1(d) = \sigma \frac{\pi d^2}{4L}, \tag{1}$$

with σ the bulk electrolyte conductivity. The value for σ is assumed to be 7.5 S m^{-1} in 1 M LiCl, or 10.5 S m^{-1} in 1 M KCl, consistent with values measured with a conductivity meter and reported in the literature elsewhere. The model (1) matches the conductance vs. nanopore diameter data reported in [1] only when $d/L < 3/4$, with the data derived in 1M KCl using $L = 20$ nm thin membranes and pores made using a focused electron beam and subsequently imaged by TEM. Nonetheless, the model is simple to manipulate and unambiguous in the calculation for the diameter for any measured value for G:

$$d_1 = \sqrt{\frac{4GL}{\pi \sigma}}, \tag{2}$$

with the subscript used to match the estimated diameter with the corresponding model. Based on the results in [1], this is assumed to be an appropriate model when $d < 3L/4$.

The second model incorporates the effect of access resistance and matches the data for all reported d/L values (i.e., smaller and larger than 1), provided the membrane thickness L is replaced by an effective thickness $l = L/\alpha$, $\alpha \geq 1$, heuristically as a way to accommodate for thinning in the membrane at the nanopore. The model is:

$$G_2(d, l) = \sigma \frac{\pi d^2}{4l} \left[\frac{1}{1 + \frac{\pi d}{4l}} \right]. \tag{3}$$

This model can be solved for d to estimate the diameter from any measured value for G; however, d will also depend on the choice for l. The only non-negative solution is:

$$d_2(l) = \frac{G}{2\sigma} + \sqrt{\left(\frac{G}{2\sigma}\right)^2 + \frac{4lG}{\pi\sigma}}. \tag{4}$$

With $l = L/\alpha$, the estimated diameter depends on the choice for $\alpha \geq 1$. In [1], the value $\alpha = 20/8.6 \approx 2.3$ was found by least-squares to fit data for $5 \leq d \leq 100$ nm with $L = 20$ nm. Other studies use different values for α. For 2 nm pores formed using dielectric breakdown in 10 nm thin SiN membranes, $\alpha = 1$ is used [2]. With 3 nm pores formed in 7 nm thin membranes using TEM [3], and for 3-4 nm pores formed in 4-5 nm thin membranes using a Helium Ion Microscope [4], the same value $\alpha = 3$ best matched the data. Inferring the shape of the pore, from conductance and TEM imaging data, has been a part of other studies [5, 6].

As in [1], we show here a comparison between $G_1(d)$ and $G_2(d, L/\alpha)$ for $\alpha = 1, 2$ and 3 while varying d (Fig. S1a), which shows that the models are relatively consistent for $d/L < 3/4$, while for $d/L > 1$ the model (3) is chosen as it is superior in matching experimental data (Fig. 1 in [1]). For a given range of recorded G over the time course of an experiment, one can determine which model is more appropriate by comparing the range to the modeled values; however, the choice for α, while heuristic, has a significant effect on the estimated d_2 value. For the range 59-63 nS for example (Fig. S1b), the pore diameter at the start of the experiment (59 nS) can be estimated to be as small as 14.5 nm when $\alpha = 3$, or as large as 21.5 nm when $\alpha = 1$. The evolution of of the inter-event conductance over a 30 minute experiment with DNA is shown in Fig. S1c, and the computed diameter models using this conductance time history is shown in Fig. S1c using d_1 from (2) and d_2 from (4), comparing $\alpha = 1$ and 3. In all cases, the estimated pore size increased by less than 0.5 nm over 30 minutes. However, the estimated values vary by up to 3 nm between the models. We also note that there is variability in the precise value for L, but this is beyond the scope of this study, and we simply assume the vendor (Norcada) delivers 30 nm ± 2 nm as promised.

The value for $G = I/V$ over the course of an experiment typically increases slowly over time. If there is an observed increase, there are two potential sources. First, the nanopore can enlarge over time, permitting an increasing amount of current to flow; this occurs at a higher rate for pores that are less "stable" (i.e., pores in membranes that are fragile for one reason or another, or that can grow since the membrane is very thin and/or due to the application of higher voltages that can etch away the membrane material). Second, the increase may be due to evaporation of water and a commensurate relative increase in the ion concentration in the "open" chamber above the nanopore to which reagents are added. One can test which of these sources is in play by replacing the buffer in the exposed chamber and remeasuring the conductance - if the value returns to the original value, the pore size and shape has likely remained in tact; if the value is higher, the pore has likely enlarged. Our results included data for which the pore measurably enlarged in the some cases, and for which it did not measurably enlarge in other cases. Our setup exhibits a small amount of evaporation, and the fluidic interface to the pore can be engineered to eliminate this effect entirely.

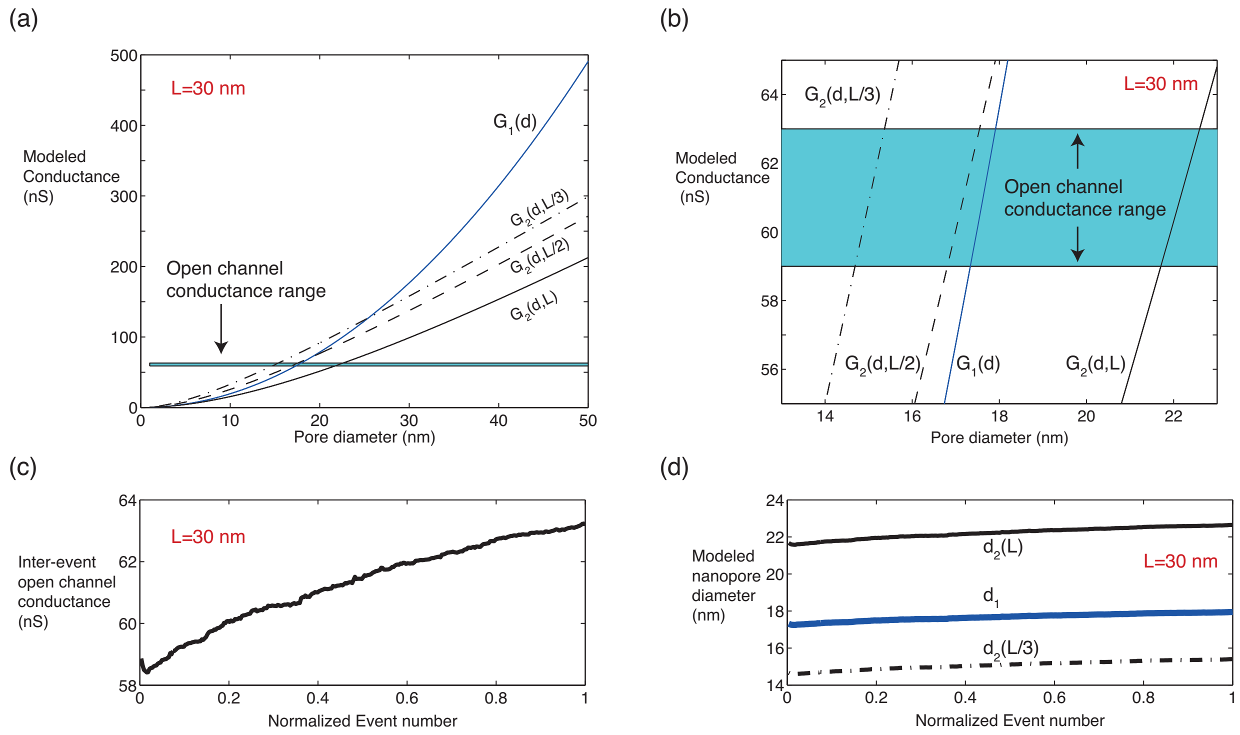

Figure S1: **Modeling nanopore diameter from conductance measured over time.** (a) Model $G_1(d)$ in (1) and $G_2(d, l)$ in (3) are comparable for $d/L < 3/4$, whereas $G_2(d, l)$ is the better model for larger d/L. (b) At any conductance values or within any given conductance range, changing l in $G_2(d, l)$ results in a different diameter d. (c) Evolution of the mean inter-event conductance using a 30 minute experiment with DNA (324 bp DNA, 2 nM, 100 mV, 1M LiCl, 322 events). (d) Evolution of the model predicted nanopore diameter, from the conductance in (c) using d_1 from (2) and d_2 from (4) with $\alpha = 1$ and 3.

In summary, using known L and $d = d_1$ from (2), if $d/L < 3/4$ we assume the model $G_1(d)$ in (1) is correct and compute d_1 from (2). Otherwise, we assume $G_2(d, l)$ in (3) is correct and compute $d_2(l)$ from (4) to estimate the pore diameter. For the model (4), we use $l = L$ for pores fabricated using a Helium Ion Microscope (HIM), since other work suggests a more columnated pore as it enlarges [5], and $l = L/3$ for pores fabricated using dielectric breakdown with which we assume a more hour glass shape is produced.

Comparing modeled diameters in a DNA and DNA/bisPNA experiment

In the DNA and DNA/bisPNA experiment compared in the main text Figure 3, we compare 324 bp DNA alone and the same DNA with a single 7 bp bisPNA bound in the middle. Figure S2 shows the evolution of the mean inter-event conductance (top) and the estimated nanopore diameter using d_1 from (2) (bottom). For the epochs (i-vi) of varying duration shown, the increase in conductance over time suggests the pore is enlarging slowly (0.7 nm over 210 minutes total). Brief periods of conductance decrease in the initial portion of

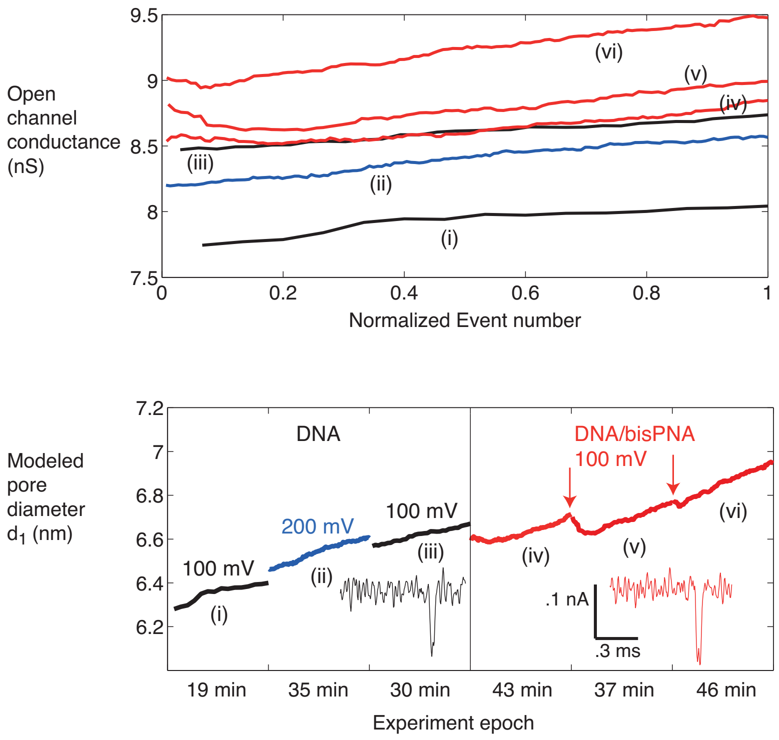

Figure S2: **Conductance measured over time (top) is used to estimating the nanopore diameter (d_1 from (2), bottom figure) for a single experiment.** Experiment epochs include: 10 nM 324 bp DNA at (i) 100 mV, (ii) 200 mV, then (iii) 100 mV; perfusion of the chamber; and three epochs (iv-vi) of DNA with a single bisPNA bound at 100 mV, with perfusion of the chamber separating each epoch (see main text Figure 3 and related text). Note that the horizontal axis in the bottom plot is not uniformly scaled.

epochs (iv,v) are consistent with reducing a small amount of evaporation when the system is perfused prior to the addition of each new complex concentration. As shown in the main text Figure 3, the DNA and DNA/bisPNA events are indistinguishable for this nanopore, primarily because for each molecule type the complex passes too fast through the pore to resolve the amplitude.

Comparing modeled diameters in two DNA/bisPNA experiments

In the two DNA/bisPNA experiments compared in the main text Figure 4, we first compare the model estimated nanopore diameters, and then discuss the noise performance and duration of the two pores. The models estimate the pore in the 10 nm membrane to have a probable diameter of 5.8-6.5 nm (thus, referenced as ∼6 nm diameter in main text), and the

pore in the 30 nm membrane to have a probable diameter of 6.5-7.5 nm (thus, referenced as ∼7 nm in main text). These ranges are shown in Fig. S3 and explained here. Both pores satisfy $d/L < 3/4$ and are initially comparable in diameter (∼6.5 nm) according to the model d_1 from (2), with the pore in the 30 nm membrane drifting to 7 nm after 2 hours of recording (an extremely stable pore). Although $d/L < 3/4$, we can estimate a range by also computing $d_2(l)$ from (4). For reasons stated above, we computed $d_2(L)$ to estimate the HIM pore in the 30 nm membrane, and $d_2(L/3)$ to estimate the dielectric breakdown pore in the 10 nm membrane. Collectively, these diameters provide the probable diameter ranges reported in Figure 4 of the main text.

The pore in the 10 nm membrane was noisier than the 30 nm membrane, for a few reasons. Noise was quantitated by computing the standard deviation of the open channel current between every pair of events, and examining the distribution. The open channel standard deviation distribution had a peak at 32 pA for the 10 nm membrane and 13 pA for the 30 nm membrane. The primary reason was the increase in effective bandwidth (55 kHz for 10 nm membrane and 30 kHz for the 30 nm membrane). Another reason is that a higher voltage was applied (200 mV) with the 10 nm membrane than the 30 nm membrane (100 mV). The third reason is that the thinner membrane results in a higher membrane capacitance, which in turn increases the amount of high frequency noise. Note too that the pore in the 10 nm membrane, formed by dielectric breakdown, also produced many more events in a short period (767 events, 23 minutes).

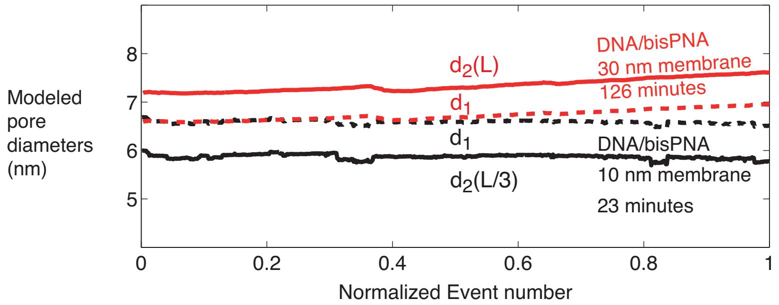

Figure S3: **Comparing estimated nanopore diameters for two separate experiments.** Models use d_1 from (2) (dashed) and d_2 from (4) (solid) with 324 bp DNA with a single bisPNA (see main text Figure 3 and related text). The lines depict the upper and lower boundary of a prospective range for the true diameter, with the 10 nm membrane (black) assumed to have an hour glass pore formed by dielectric breakdown ($d_2(L/3)$) and the 30 nm membrane (red) assumed to have a more columnated pore formed by HIM ($d_2(L)$).

References

[1] Stefan W Kowalczyk, Alexander Y Grosberg, Yitzhak Rabin, and Cees Dekker. Modeling the conductance and DNA blockade of solid-state nanopores. *Nanotechnology*, 22(31):315101, July 2011.

[2] Kyle Briggs, Harold Kwok, and Vincent Tabard-Cossa. Automated fabrication of 2-nm solid-state nanopores for nucleic acid analysis. *Small*, 10(10):2077–2086, May 2014.

[3] M Wanunu, T Dadosh, V Ray, J Jin, Larry McReynolds, and Marija Drndić. Rapid electronic detection of probe-specific microRNAs using thin nanopore sensors. *Nature Nanotechnology*, 5:807–814, 2010.

[4] Autumn T Carlsen, Osama K Zahid, Jan Ruzicka, Ethan W Taylor, and Adam R Hall. Interpreting the conductance blockades of DNA translocations through solid-state nanopores. *ACS Nano*, 8(5):4754–4760, May 2014.

[5] Jijin Yang, David C Ferranti, Lewis A Stern, Colin A Sanford, Jason Huang, Zheng Ren, Lu-Chang Qin, and Adam R Hall. Rapid and precise scanning helium ion microscope milling of solid-state nanopores for biomolecule detection. *Nanotechnology*, 22(28):285310, June 2011.

[6] Yael Liebes, Maria Drozdov, Yotam Y Avital, Yaron Kauffmann, Hanna Rapaport, Wayne D Kaplan, and Nurit Ashkenasy. Reconstructing solid state nanopore shape from electrical measurements. *Applied Physics Letters*, 97(22):223105, 2010.
