# Supplementary material for: Nanopore-Based Target Sequence Detection
Source: PLoS One. 2016 May 5;11(5):e0154426. doi: 10.1371/journal.pone.0154426 (PMC4858282; doi:10.1371/journal.pone.0154426)
Supplement: S3 Section — (PDF) [file pone.0154426.s003.pdf]

Supporting Information Sections for
Morin, *et al.*, "Nanopore-based target sequence detection"

S3. Representative events from DNA/bisPNA data with 6nm pore

The events shown in Fig. S7 are from the populations shown in the main text Figure 4, correspond to the DNA/bisPNA experiment using a ∼6 nm diameter pore in a 10 nm membrane (i.e., the smaller pore). Event characteristics (δG, duration) are reported in Table S1. We selected 17 of the first 35 events to show the most common event patterns observed across all 767 events. Event patterns are categorized qualitatively here, referencing the corresponding event number in parentheses as labelled in Fig. S4 and listed in Table S1. First, there are fast and shallow events that are consistent with unbound DNA (Nos. 11,22), along with longer lasting events at a comparable amplitude depth (Nos. 5, 20,30) which may or may not be unbound DNA. Next, there are significantly longer lasting events ($> 400\mu$s) with a single and sustained amplitude plateau (Nos. 1,4,7) which are very common. Note that the duration scale for each event in Fig. S4 is different; they were normalized by width to fit within a common frame size for visual purposes, with duration values reported in Table S1. There are also are large percentage of events with a brief initial higher amplitude shoulder followed by a lower amplitude level (Nos. 2,3,10,25,28), with a few showing the reverse of that pattern (No. 18). Finally, still others have a high-low-high pattern (No. 35, and event 75 not shown here but in main text Figure 4d). Visually screening for multi-level event patterns, we see more events (right two columns, Fig. S4) with several displaying the high-low-high pattern as for event 35. It is quite possible the two level events (Nos. 2,3,10,18,25,28) are simply high-low-high events for which the initial or final high amplitude is not resolved at the recording bandwidth. Approximately 20% of all events have at least two of these three levels. There is variation in the amplitude of the levels, with initial or final levels higher than the DNA alone levels likely a result of initial "docking" of the molecule on the pore prior to actual capture, which affect the access resistance outside the pore [1, 2]. In this paper, we do not attempt to quantitate or model specific amplitude levels within multi-level events, but focus solely on the grosser detectable differences in populations as reagents are varied.

A significant portion of these events hit an amplitude depth that would not occur for an unbound dsDNA going through a 6 nm pore at 200 mV in 1M LiCl. Specifically, Figure S5 shows the maximum δG value for all recorded events, which is a better indicator of the largest amplitude depth within each event. From [3], the model $G_1(d)$ (equation (2) in Section S2) predicts an amplitude depth of 2.85 nS for unfolded dsDNA (2.2 nm diameter), while the model $G_2(d, L/3)$ (equation (3) in Section S2) predicts an amplitude depth of 2.57 nS for unfolded dsDNA. Of the 767 recorded events, 40% (313) have a maximum δG larger than

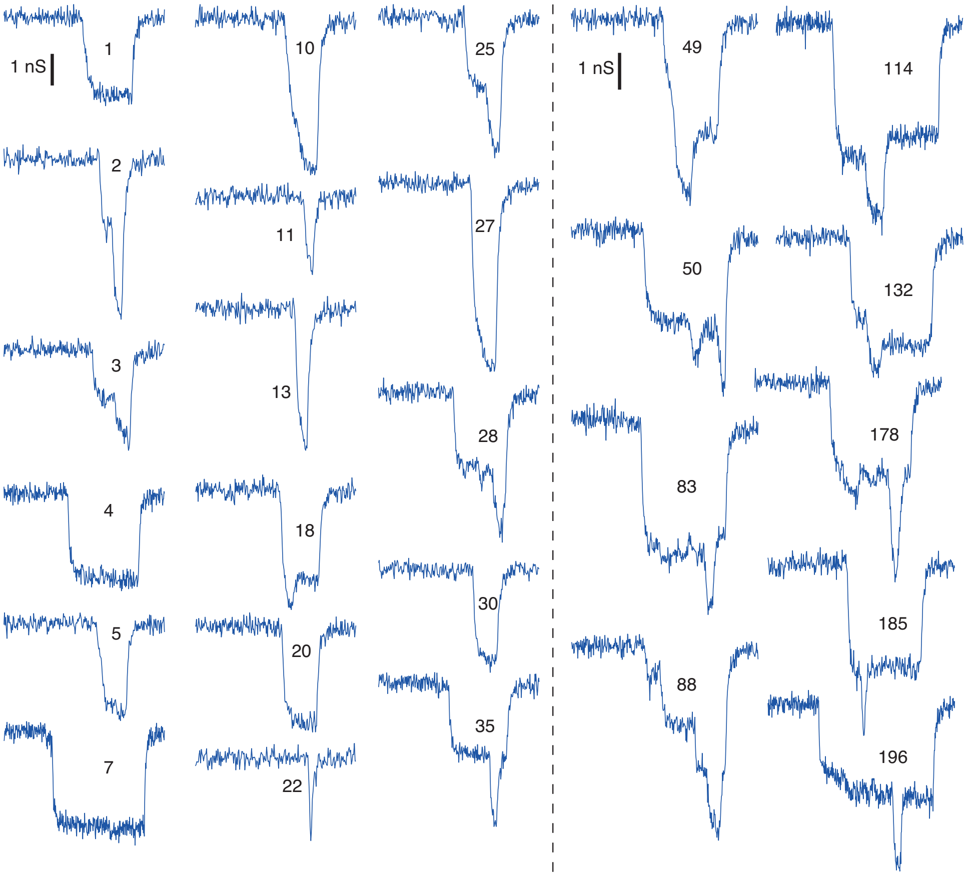

Figure S4: **Representative events from a DNA/bisPNA experiment using a ∼6 nm diameter pore in a 10 nm membrane.** Recorded 767 total events at 200 mV. The left 3 columns show 17 of the first 35 events (enumerated in order of occurrence), chosen to show the most common event patterns. The right 2 columns show more examples of the high-low-high event pattern as seen for event number 35. All events are from the experiment displayed in main text Figure 4. The δG and duration values for the 3 left columns are reported in Table S1. A common vertical scale is used for all events, with a variable duration scale used to fit events within a common width for ease of display.

2.85 nS. Since this DNA is likely too short for folded passage through the pore, and since the bis-PNAs are too small (∼17 bp equivalent) and positively charged to result in detectable signals, we attribute the deeper signals to bisPNA-bound DNA.

References

[1] Autumn T Carlsen, Osama K Zahid, Jan Ruzicka, Ethan W Taylor, and Adam R Hall. Interpreting the conductance blockades of DNA translocations through solid-state nanopores. *ACS Nano*, 8(5):4754–4760, May 2014.

Table S1: **Data for the events (up to number 35) shown in Fig. S4†.**

Event No.	δG (nS)	Duration (μs)	Event No.	δG (nS)	Duration (μs)	Event No.	δG (nS)	Duration (μs)
1	2.16	414	10	3.26	266	25	2.53	286
2	2.63	190	11	1.61	74	27	3.84	234
3	1.90	278	13	2.79	118	28	2.56	462
4	2.54	698	18	2.61	326	30	2.38	186
5	2.09	226	20	2.77	258	35	1.60	820
7	2.92	1190	22	1.33				

† Events are ordered top to bottom, and left column to right.

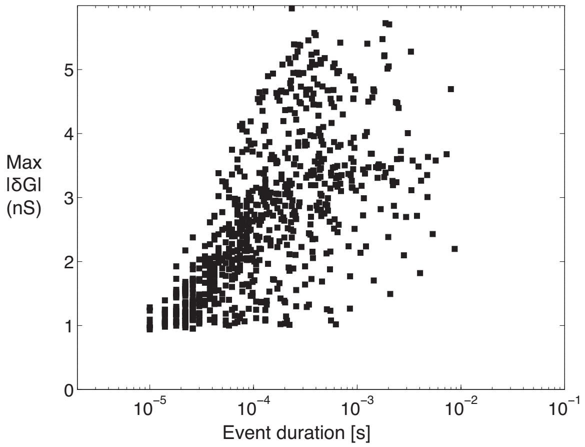

Figure S5: **Maximum δG vs. duration for all events in the DNA/bisPNA experiment using the ∼6 nm diameter pore in a 10 nm membrane at 200 mV.**

[2] Jacob K Rosenstein, Meni Wanunu, Christopher A Merchant, Marija Drndić, and Kenneth L Shepard. Integrated nanopore sensing platform with sub-microsecond temporal resolution. *Nature Methods*, 9(5):487–492, March 2012.

[3] Stefan W Kowalczyk, Alexander Y Grosberg, Yitzhak Rabin, and Cees Dekker. Modeling the conductance and DNA blockade of solid-state nanopores. *Nanotechnology*, 22(31):315101, July 2011.
