# Supplementary material for: Nanopore-Based Target Sequence Detection
Source: PLoS One. 2016 May 5;11(5):e0154426. doi: 10.1371/journal.pone.0154426 (PMC4858282; doi:10.1371/journal.pone.0154426)
Supplement: S4 Section — Representative events from DNA/bisPNA and DNA/bisPNA-PEG data sets are compared. (PDF) [file pone.0154426.s004.pdf]

Supporting Information Sections for
Morin, *et al.*, "Nanopore-based target sequence detection"

S4. Comparing DNA/bisPNA and DNA/bisPNA-PEG with 17-21 nm pore

In the DNA-PNA-PEG (5 kDa) experiment compared in the main text Figure 5, we compare 324 bp DNA with a single 7 bp bis-PNA bound in the middle, to that same complex but with up to 3 PEG (5 kDa) molecules attached to each PNA. Figure S6 shows the evolution of the mean inter-event conductance (top) and the estimated nanopore diameter using d_1 from equation (2) in Section S2 (bottom). For the epochs (i-vi) of varying duration shown,

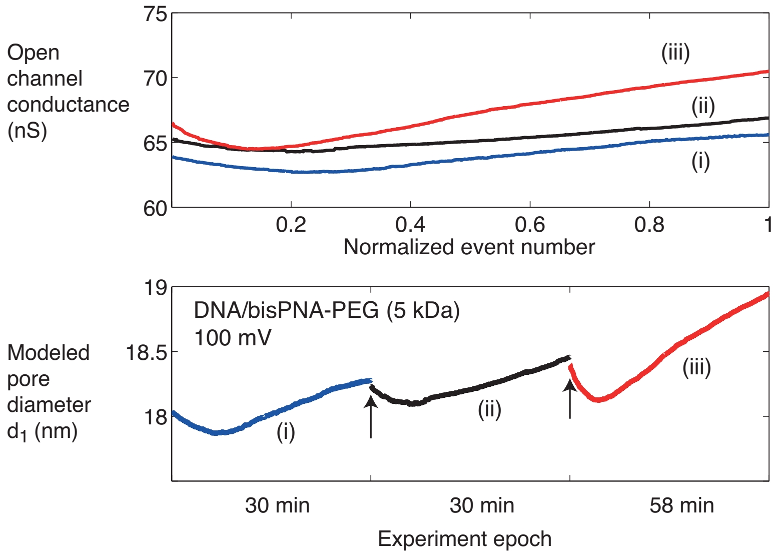

Figure S6: **Conductance measured over time (top) and estimated nanopore diameter (bottom) for an experiment with DNA/bisPNA-PEG 5 kDa.** Modeled diameter d_1 is from equation (2) in Section S2, with 2 nM 324 bp DNA/bisPNA-PEG 5 kDa at 100 mV in 1M LiCi. Three epochs (i-iii) were recorded with fresh complex added at the start of each epoch, and with adjacent epochs separated by 2X perfusion of the chamber (up arrows). Note that the horizontal axis in the bottom plot is not uniformly scaled.

the increase in conductance over time suggests the pore is enlarging slowly (~1 nm over 2 hours). Conductance decrease (< 1 nS, or 1.5%) in the initial period of each epoch is consistent with reducing the small amount of evaporation that occurs over each epoch.

Representative events from DNA/bisPNA and DNA/bisPNA-PEG experiments

The events shown in Fig. S7 are from the populations shown in the main text Figure 5. Event characteristics (δG, duration) are reported in Table S2.

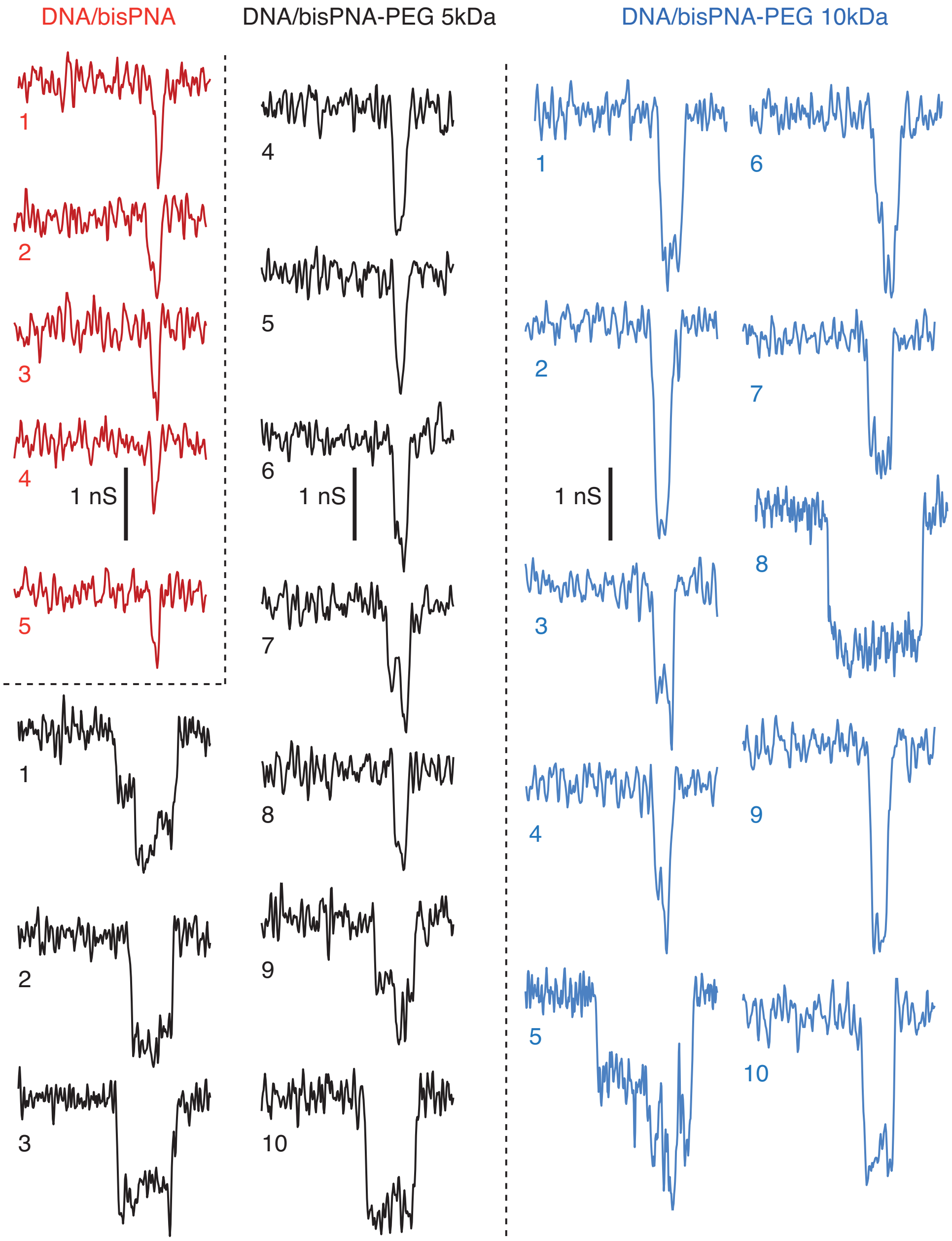

Figure S7: **Representative events from the experiment displayed in main text Figure 5.** The δG and duration values are reported in S2 Table, with a common vertical scale for all events but a variable duration scale used to fit all events within a common width.

Table S2: **Data for DNA/bisPNA and DNA/bisPNA-PEG events shown in Fig. S7†.**

Event* No.	δG (nS)	Duration (μs)	Event* No.	δG (nS)	Duration (μs)	Event* No.	δG (nS)	Duration (μs)
DP-1	0.77	28	DP5P-1	1.27	380	DP10P-1	1.83	120
DP-2	0.80	56	DP5P-2	1.43	232	DP10P-2	1.88	132
DP-3	1.00	24	DP5P-3	1.24	364	DP10P-3	1.51	92
DP-4	1.03	40	DP5P-4	1.44	68	DP10P-4	1.49	88
DP-5	0.84	32	DP5P-5	1.27	56	DP10P-5	1.60	820
			DP5P-6	1.42	64	DP10P-6	1.76	124
			DP5P-7	1.15	104	DP10P-7	1.61	116
			DP5P-8	1.16	56	DP10P-8	1.88	792
			DP5P-9	1.08	204	DP10P-9	2.23	100
			DP5P-10	1.53	296	DP10P-10	1.94	156

* DP-i = DNA/bisPNA experiment event number i, DP5P-i = DNA/bisPNA-PEG (5 kDa) experiment and event number i, and DP10P-i = DNA/bisPNA-PEG (10 kDa) experiment and event number i. The event number i matches the number shown near each event in Fig. S7.
† Events are ordered top to bottom, and left column to right.

The noise performance of the nanopore was consistent for all three reagents, as shown in Figure S8.

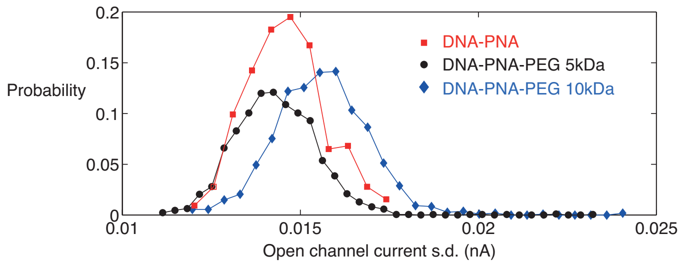

Figure S8: **The noise performance of the nanopore was consistent for all three reagents tested in Fig 5.** Histogram of the standard deviation of the open channel current between every pair of events, for the three data sets shown in S7 Fig and main text Fig 5.
