# Supplementary material for: Nanopore-Based Target Sequence Detection
Source: PLoS One. 2016 May 5;11(5):e0154426. doi: 10.1371/journal.pone.0154426 (PMC4858282; doi:10.1371/journal.pone.0154426)
Supplement: S5 Section — (PDF) [file pone.0154426.s005.pdf]

Supporting Information Sections for
Morin, *et al.*, "Nanopore-based target sequence detection"

S5. Larger pore (36 nm) experiment with DNA/bisPNA-PEG 10 kDa

An experiment with 2 nM DNA/bisPNA-PEG (10 kDa) was conducted in a larger pore, estimated to be ∼36 nm in diameter in a 30 nm membrane, using 100 mV in 1M LiCl. Since these are the same reagent concentration, voltage and buffer conditions used in the DNA/bisPNA-PEG 10 kDa data shown in the main text Figure 5, we can compare the event distributions to examine the effect of increasing the nanopore size on these distributions. The smaller pore was modeled to be 20-21 nm (main text Figure 5) using d_1 from equation (2) of Section S2, but is ∼27 nm using the $d_2(L)$ model from equation (4) of Section S2. As described previously, the two models are comparable provided $d/L < 0.75$, which is the case when $d < 22.5$ nm for the 30 nm membranes used. Note that no model of the nanopore diameter is exact, and the true diameter and shape cannot be identified without more complex analysis and measurements, which are beyond the scope of this study. Moreover, although the two models differ in the estimated nanopore size, it is not important that we know exactly the nanopore size; instead, an approximate size estimate is sufficient, as it allows to make approximate size comparisons with the size of the complexes we are testing, and comparisons between nanopores. For the pores larger than $d > 22.5$ nm, we use $d_2(L)$ as the more appropriate model (see Section S2 for justification). For the two nanopores, the $d_2(L)$ model predicts a 27 nm diameter for the smaller pore and a 36 nm diameter for the larger pore. The noise of the larger nanopore was modestly higher, as shown in Figure S9.

Prior to testing DNA/bisPNA-PEG (10 kDa) complexes with the larger pore, a set of negative controls were run. First, buffer only was tested for 42 minutes producing 4 flagged events (presumed to be aperiodic noise). Subsequently, 2 nM bisPNA-PEG (5 kDa) was tested for 34 minutes producing 13 events, followed by 2 nM PEG alone (10 kDa) producing only 2 events over 27 minutes. All of these flagged events in negative control experiments are more likely aperiodic noise than actual translocations, nearly all of which are fast "spikes" in appearance with a duration below 24 μsec. Such aperiodic events are commonly observed by others, earning the name " ghost events" [1] and "bouncing spikes" [2], and are usually thought to be noise (in buffer only conditions) or collisions of molecules with the pore (in the presence of molecules) or potentially both, though it appears impossible to determine which is the cause in each case or to rule out other potential causes (e.g., inhomogeneity in the membrane material transiently reacting to the voltage potential and causing current signal variations related to capacitance changes/re-charging). Since PEG is neutrally charged in the

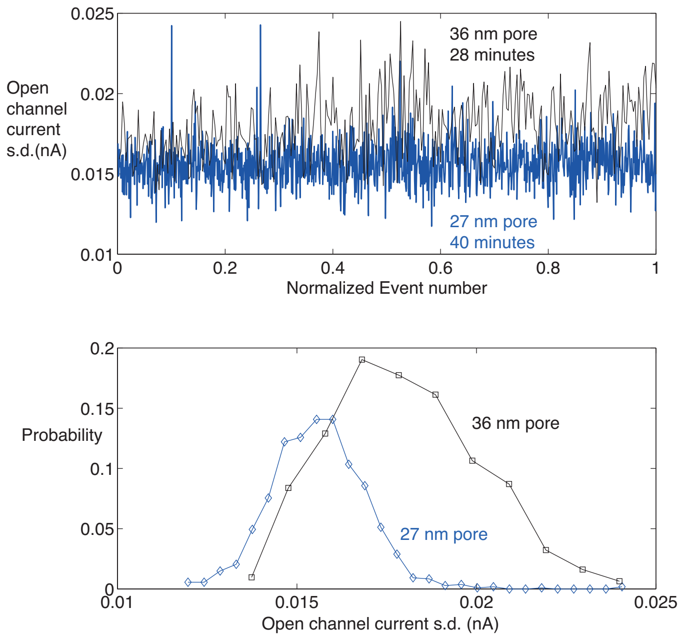

Figure S9: **Comparing noise between two nanopores used to measured DNA/bisPNA-PEG 10 kDa**. (Top) Evolution of the standard deviation of the open channel current, computed between every pair of events, and corresponding histogram (bottom) for the two nanopore sizes (30 nm membranes, 100 mV, 1M LiCl). The larger pore has a higher peak (17-18 pA) and larger spread in the histogram.

buffer used and thus unlikely to be capturable in the nanopore by voltage, and bisPNA adds little size to a bisPNA-PEG, any free bisPNA-PEG or PEG alone are unlikely to produce the deeper and longer lasting events produced with the full complex present in the chamber above the pore. Moreover, negative control DNA (324 bp without the full 7 bp binding sequence, main text Figure 2) does not bind the bisPNA alone or bisPNA-PEG, further suggesting the full complex alone produced the deeper and longer lasting events observed.

Following the PEG alone and bisPNA-PEG negative controls, 2 nM DNA/bisPNA-PEG (10 kDa) was added, producing 310 events over 28 minutes at a capture rate of 0.28 1/sec (R^2 = 0.997). Representative events and a comparison between event populations and estimated nanopore size are shown in Figure S10, comparing the data set to a set recorded with the smaller pore (main text Figure 5). As expected, the same size molecules produce shallower events (i.e., smaller δG) in the larger pore. In particular, while 10.98% (118) of events satisfied $\delta G > 1.5$ nS and duration longer than 24 μsec with the smaller pore, 2.58% (8) of events met that criteria with the larger pore. Meanwhile, the duration distribution was conserved, with the number of events with a duration longer than 60 μsec at 18.88% (203) for the 27 nm pore and 20.32% (63) for the 35 nm pore. In terms of summary statistics, the duration values were the same for both pores (median=24 μsec, IQR =36 μsec), with the 27 nm pore had a higher mean δG (1.11 $\pm$ 0.3 nS) than the 36 nm pore (1.05 $\pm$ 0.2 nS), again, as expected. Observing a decrease in δG with increasing pore size for a given complex is

consistent with other nanopore assays [3].

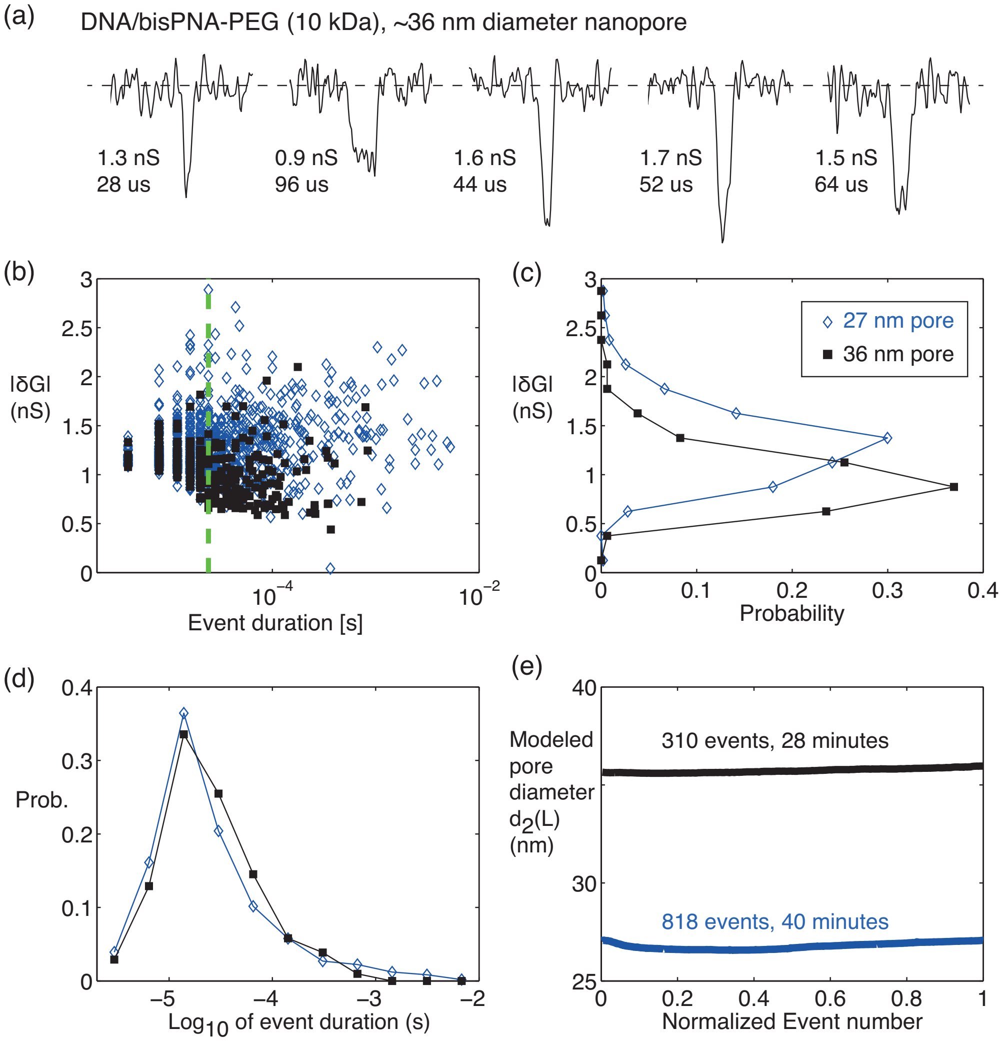

Figure S10: **The DNA/bisPNA-PEG 10 kDa complex remains resolvable with increasing pore size up to 36 nm in diameter.** (a) Representative events with the larger pore reporting δG and duration values. (b) Population of δG vs. duration for all events in each data set, each at 2 nM and 100 mV in 1 M LiCl, with the green line (24 μsec) the duration minimum for resolving δG. (c) δG histogram and (d) duration histogram. Only the δG histogram appears shifted by the increased pore size. (e) Evolution of the modeled nanopore diameter, using the time history of the inter-event open channel conductance.

References

[1] Calin Plesa, Justus W Ruitenberg, Menno J Witteveen, and Cees Dekker. Detection of Individual Proteins Bound along DNA Using Solid-State Nanopores. *Nano Letters*, May

2015.

[2] Jae-Seok Yu, Min-Cheol Lim, Duyen Thi Ngoc Huynh, Hyung-Jun Kim, Hyun-Mi Kim, Young-Rok Kim, and Ki-Bum Kim. Identifying the Location of a Single Protein along the DNA Strand Using Solid-State Nanopores. *ACS Nano*, May 2015.

[3] Stefan W Kowalczyk, Alexander Y Grosberg, Yitzhak Rabin, and Cees Dekker. Modeling the conductance and DNA blockade of solid-state nanopores. *Nanotechnology*, 22(31):315101, July 2011.
