# Supplementary material for: Nanopore-Based Target Sequence Detection
Source: PLoS One. 2016 May 5;11(5):e0154426. doi: 10.1371/journal.pone.0154426 (PMC4858282; doi:10.1371/journal.pone.0154426)
Supplement: S6 Section — (PDF) [file pone.0154426.s006.pdf]

Supporting Information Sections for
Morin, *et al.*, "Nanopore-based target sequence detection"

S6. Largest pore (50 nm) experiment with DNA/bisPNA-PEG 10 kDa

An experiment with DNA/bisPNA-PEG (10 kDa) was conducted in a pore estimated to be ~50 nm in diameter using $d_2(L)$ from equation (4) of Section S2 (30 nm membrane). The complex was titrated in three epochs from 10 nM to 5 nM and then 2 nM, all at 90 mV in 1M LiCl. To keep the gain setting that produces the lowest noise in the 700B amplifier, the voltage was set to 90 mV rather than 100 mV to keep the current within ±20 nA at 1M LiCl. Figure S11 shows the evolution of the mean inter-event conductance (top) and the estimated nanopore diameter using (bottom), comparing both conductance and estimated diameter to the smaller nanopore data with DNA/bisPNA-PEG (10 kDa) described in the main text (Figure 5).

The noise of this largest pore was quite high, compared to the other pores tested. Specifically, the noise of the 50 nm pore had a peak of 22 pA for the histogram of the open channel current standard deviation (not shown), compared to 16-18 pA for the 27 nm and 36 nm diameter pores (Fig. S9). The larger pore also displayed are higher than normal false event rate (attributed to aperiodic noise spikes). Specifically, buffer only for 67 minutes produced 47 flagged events (0.017 1/sec rate). Nonetheless, when the full complex was added, the event rate increased significantly, generating 956 events over 56 minutes (0.32 1/sec, $R^2 = 0.997$) at 10 nM complex. Perfusing the chamber and adding a lower concentration (5 nM) of complex resulted in 713 events over 53 minutes (0.25 1/sec rate, $R^2 = 0.998$). Perfusing the chamber and adding 2 nM complex resulted in 543 events over 47 minutes (0.2 1/sec rate, $R^2 = 0.999$). The event distributions and dependence of capture rate on concentration data are shown in Figure S12. The modeled pore size remained ~50 nm for all three epochs, suggesting the pore was stable and essentially columnated, while observed drift in modeled pore size was caused by slow evaporation and not a change in the actual pore geometry, as described in an earlier section. Since the noise was larger for the 50 nm pore, and since a larger percentage of faster and shallower events go undetected with the larger pore, it did not make sense to do a detailed comparison between the DNA/bisPNA-PEG 10 kDa event populations with the other smaller pore sizes.

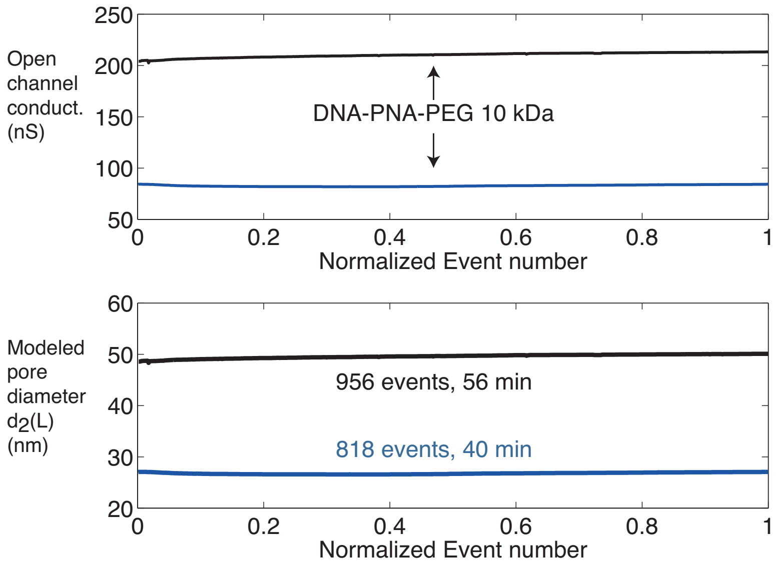

Figure S11: **Doubling the nanopore pore size in experiments with DNA/bisPNA-PEG 10 kDa.** Conductance measured over time (top) is used to estimate the nanopore diameter $d_2(L)$ (bottom) for two separate experiments with 324 bp DNA-bis-PNA-PEG 10 kDa. The smaller pore (~27 nm diameter) generated events at 100 mV and 2 nM complex concentration, while the larger pore (49-51 nm diameter) generated events at 90 mV and 10 nM complex concentration. Note that the horizontal axis is not uniformly scaled.

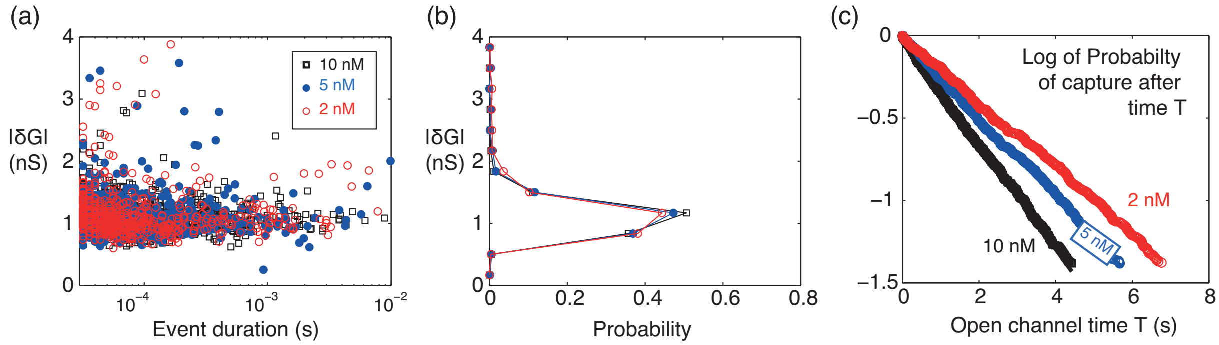

Figure S12: **DNA/bisPNA-PEG (10 kDa) are detectable with a ∼50 diameter pore in a 30 nm membrane.** (a) Population of δG vs. duration for all events in three epochs of recording with DNA/bisPNA-PEG at 10 nM, 5 nM and 2 nM, and 100 mV in 1M LiCl. (b) Event δG histogram. (c) On the natural-log scale, the fraction of open channel times faster than time T (s) for the three data sets, with a straight line fit to the data shown used to quantitate capture rates: 0.32 1/sec at 10 nM, 0.25 1/sec at 5 nM, and 0.2 1/sec at 2 nM.
