# Supplementary material for: Nanopore-Based Target Sequence Detection
Source: PLoS One. 2016 May 5;11(5):e0154426. doi: 10.1371/journal.pone.0154426 (PMC4858282; doi:10.1371/journal.pone.0154426)
Supplement: S7 Section — The subsections present the framework in detail. A final subsection details application of the framework to data in Fig 5. (PDF) [file pone.0154426.s007.pdf]

Supporting Information Sections for
Morin, *et al.*, "Nanopore-based target sequence detection"

S7. Mathematical framework for assigning statistical significance to nanopore event subpopulation detection

The goal is to identify a mathematical criterion upon which to make the call that a molecular species is present, and also to assess the statistical significance of that call. We can formulate the problem as follows. First, we identify two categories of molecules in bulk solution: type 1 are all the background molecules (e.g., unbound DNA, free PNA, free PEG-bound PNA, etc.), and type 2 are the molecules of interest (i.e, DNA/PNA-PEG complexes). Our goal is to detect the presence of type 2 molecules in bulk solution (future work will provide tools with which to estimate its concentration). An event is called type 1 or 2 if the molecule captured in that event is type 1 or 2, respectively. Based on data from control experiments, we try to identify an event signature that is almost absent in type 1 events but is present in a significant fraction of type 2 events. An event is "tagged" if the signature is present in that event. An example signature might be $\delta G > x$, for some identified threshold conductance shift x. Shifts are typically larger for larger molecules going through a pore of a given size; so, this criterion is intuitive since sequence-specific labels (PNA-PEG) bound to a DNA create larger features. Note that our formulation does not require δG to be the variable used to establish the signature; the signature can be based on any variable or set of variables used to quantitate each event in the recorded set.

We define the variables

$$q_1 = \Pr(\text{tagged} \mid \text{type 1 event}), \quad q_2 = \Pr(\text{tagged} \mid \text{type 2 event}).$$

If we view "tagging" as labeling an event as type 2, then q_1 is the false positive probability and $(1 - q_2)$ is the false negative probability. The challenge is to select an event signature such that q_1 is very small, and $q_2 - q_1 \gg q_1$ (the larger, the better). Let $p =$ the probability that a capture event is type 2, and $q(p) =$ the probability that a capture event is tagged. Probability p is related to concentrations in bulk solution as

$$p = \frac{[\text{type 2}]r_2}{[\text{type1}]r_1 + [\text{type 2}]r_2},$$

with capture rate constants $r_i, i = 1, 2$ in units of capture rate per unit concentration. The probability q that a capture event is tagged is a function of p, given by the equation:

$$q(p) = q_1 + p * (q_2 - q_1).$$

Each event is either tagged or not. Let

$$X = \begin{cases} 1, & \text{tagged} \\ 0, & \text{untagged} \end{cases}$$

X has a Bernoulli distribution with probability $q(p)$. We study the quantity

$$Q(p) = \frac{\text{Number of tagged events}}{\text{Total number of events}} = \frac{1}{N}\sum_{j=1}^{N} X_j$$

where N is total number of all events (tagged or not). The true mean of X is $q(p)$, while $Q(p)$ is a sample mean of X. Also, $Q(p)N$ has a binomial distribution with parameters N and $q(p)$. When there are no type 2 molecules in bulk solution, we have $p = 0$ and

$$Q(0) \approx q(0) = q_1$$

When there are type 2 molecules in bulk solution, we have $p > 0$ and

$$Q(p) \approx q(p) = q_1 + p(q_2 - q_1)$$

The general idea is as follows

- In a control experiment with $p = 0$, $Q(0)$ is determined with good accuracy from a large number of capture events;
- In a detection experiment with unknown p, $Q(p)$ is computed from the capture events over a prescribed time period;
- Based on the confidence interval of $Q(p)$ (which we estimate below), we decide whether or not $Q(p) > Q(0)$ is statistically sound (and thus, $p > 0$ is statistically sound).

Note that if q_1 is very small, and $q_2 - q_1 \gg q_1$, then $Q(p) > Q(0)$ can hold even with a small number of events. The key is whether $Q(p) - Q_* > Q(0)$ is still true, with Q_* the 99% (for example) confidence interval for $Q(p)$.

Estimating the confidence interval of $Q(p)$

Simplifying notation, denote probability $q = q(p)$ and random variable $Q = Q(p)$. Since QN has a binomial distribution with parameters N and q, the mean and standard deviation of Q are

$$\text{mean}(Q) = q, \quad \text{std}(Q) = \sqrt{\frac{q(1-q)}{N}}$$

Since q is unknown, we can instead use the observed (computed) value for the random variable Q (still denoted Q) to approximate q in the standard deviation of Q, given by

$$\text{std}(Q) \approx \sqrt{\frac{Q(1-Q)}{N}}$$

Normal approximation

We approximate the distribution of $Q(p)$ using a normal distribution with the same mean and variance. Let $z_{\alpha/2}$ be the critical value of the standard normal distribution for a given error level α. Then $z_{\alpha/2}$ is defined as

$$\Pr\left(Y > z_{\alpha/2}\right) = \frac{\alpha}{2}$$

where Y is a standard normal distribution (mean = 0, variance = 1). For specific confidence intervals we can quantitate $z_{\alpha/2}$, such as

- For $\alpha = 0.05$, $z_{\alpha/2} = 1.96$, which corresponds to 95% confidence interval.
- For $\alpha = 0.02$, $z_{\alpha/2} = 2.3263$, which corresponds to 98% confidence interval.
- For $\alpha = 0.01$, $z_{\alpha/2} = 2.5758$, which corresponds to 99% confidence interval.

An approximate confidence interval is

$$Q \pm z_{\alpha/2}\sqrt{\frac{Q(1-Q)}{N}}$$

Again, Q here is actually an observed value of random variable $Q(p)$ from a given experiment. For the chosen α (confidence interval),

$$Q(p) > Q(0) \quad \text{is statistically sound if} \quad Q - z_{\alpha/2}\sqrt{Q(1-Q)/N} > Q(0).$$

In Matlab, $z_{\alpha/2} = \sqrt{2} \cdot \mathrm{erfcinv}(\alpha)$. The confidence intervals reported in the main text use the Normal Approximation, though we note that the results were consistent when using the Wilson confidence interval defined below.

Wilson confidence interval

When q is small and N is not very large, the assumption of normal approximation is not valid, and a better approximation is to use the Wilson confidence interval. Using $b = z_{\alpha/2}^2/N$, the interval is given by

$$\left(\frac{Q + b/2}{1+b}\right) \pm z_{\alpha/2}\left(\frac{\sqrt{\frac{Q(1-Q)}{N} + \frac{b}{4N}}}{1+b}\right) \tag{1}$$

For the chosen α (confidence interval),

$$Q(p) > Q(0) \quad \text{is statistically sound if} \quad \left(\frac{Q + b/2 - z_{\alpha/2}\sqrt{\frac{Q(1-Q)}{N} + \frac{b}{4N}}}{1+b}\right) > Q(0).$$

We note that one could also use the Clopper-Pearson interval, or the P-value method of hypothesis testing.

Estimating the minimum N and recording time T to achieve $q(p) > q_1$

This section provides an approximate method for identify the minimum number of events required to assert that $q(p) > q_1$. Consider the case that q_1 is known and small and $q(p) > q_1$. Then with probability >99%, the observed value of Q that approximates $q(p)$ will lead us to $Q > q_1$ when

$$(Q - q_1) \geq [4 \text{ times standard deviation of } Q]$$

which depends on N. Disclaimer: this is a ballpark estimate, which is easy to work with analytically. The number of events needed satisfies

$$Q - q_1 \geq 4\sqrt{\frac{Q(1-Q)}{N}}$$

After algebra, this results in

$$N \geq 16\frac{Q(1-Q)}{(Q-q_1)^2}$$

For example, assuming the values $q_1 = 0.5\%$ and $Q = 6\%$, the equation above suggests $N \geq 300$, a reasonable value. From this expression we can also estimate the time T needed to achieve 99% confidence. If the total capture rate is k_c, the time needed is

$$T = \frac{N}{k_c} = \frac{16}{k_c} \cdot \frac{Q(1-Q)}{(Q-q_1)^2}.$$

The value for T is an estimate for the first time to 99% confidence detection, which can serve as a time-to-results metric for nanopore assays.

Application of Mathematical Framework to data in Figure 5

As described in the main text, we considered DNA/bisPNA-PEG complexes as the molecules of interest ("type 2") that signal the presence of a target sequence, utilizing the data shown in Figure 5. The nanopore initially tested PEG alone and DNA/bisPNA prior to measuring DNA/bisPNA-PEG, and the DNA/bisPNA data was treated as a negative control ($Q(0) = 1.21\%$) based on using a minimum duration of 50 μsec as the tagging criteria. An error-bar plot of $Q(p) \pm Q_*$ as a function of recording time is shown in Figure S13. The values for $Q(p)$ and Q_* are updated at each point in time when a new event is detected. The $Q(p) \pm Q_*$ trend converges to $25.9 \pm 3.95\%$ for DNA/bisPNA-PEG 10 kDa and to $6.09 \pm 0.95\%$ for DNA/bisPNA-PEG 5 kDa.

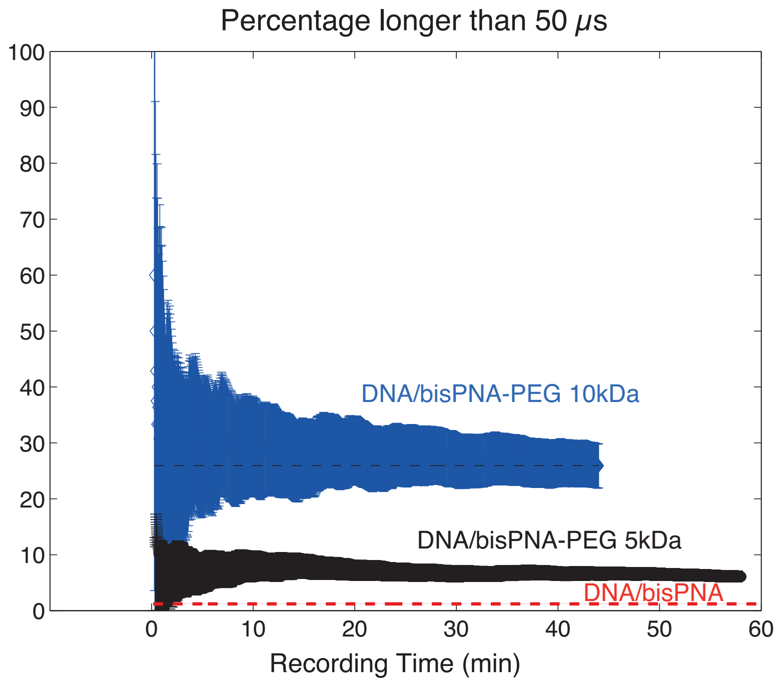

Figure S13: **Evolution of $Q(p) \pm Q_*$ as a function of recording time for the DNA/bisPNA-PEG 5, 10 kDa data in Fig 5.** The envelope width shows the evolution and attenuation of the uncertainty over time, and as compared to the false-positive threshold ($Q(0) = 1.21\%$) established from the DNA/bisPNA data (red dashed line).

We also examined the margin of robustness of the positive detection result shown in Figure S13. Specifically, a quantitative test of robustness is to compute the range of criteria threshold value(s) that preserve the 99%-confidence detection result, using all events that were recorded for each data set. Using the data from Figure 5, a plot comparing $Q(p) - Q_*$ for DNA/bisPNA-PEG (5,10 kDa) and $Q(0)$ for DNA/bisPNA was generated while varying the duration threshold T_{cut} (μs) used to tag events as type 2 (Fig. S14). Every duration threshold T_{cut} for which the $Q(p) - Q_*$ line exceeds the $Q(0)$ in Figure S14a is a positive result (99%-confidence detection). Equivalently, Figure S14b shows the T_{cut} range over which $Q(p) - Q_* - Q(0) > 0$. The trends show that 99% detection confidence in preserved for any duration threshold in the ranges $[28, 2900]\mu$s for DNA/bisPNA-PEG (5 kDa) and $[12, 4600]\mu$s for DNA/bisPNA-PEG (10 kDa). Observe the T_{cut} values that maximize $Q(p) - Q_* - Q(0)$ in Figure S14b provide the most robust choice.

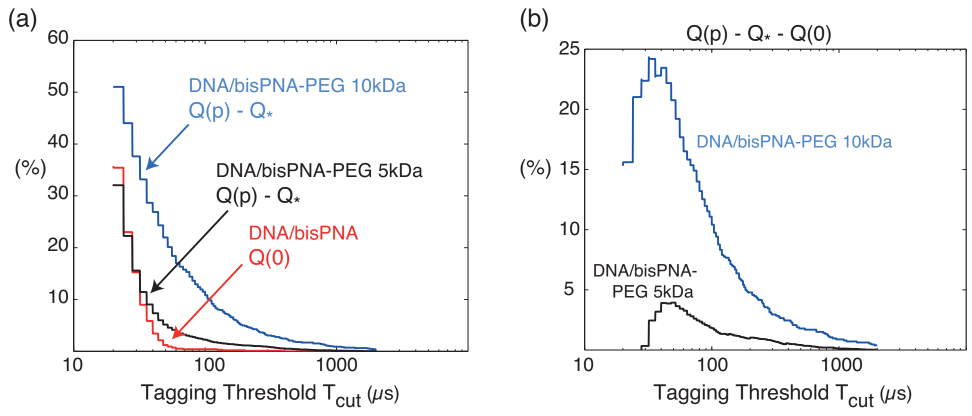

Figure S14: **Examining the margin of robustness for positive detection using a minimum duration threshold**. (a) $Q(p) - Q_*$ for the DNA/bisPNA-PEG 5, 10 kDa data and $Q(0)$ for the DNA/bisPNA data in the main text Fig 5, using all events in each case, and while varying the minimum duration T_{cut} (μs) for tagging events. (b) The T_{cut} range over which $Q(p) - Q_* - Q(0) > 0$ (using same data in (a)), and thus over which 99% detection confidence is achieved.
