# Supplementary material for: Nanopore-Based Target Sequence Detection
Source: PLoS One. 2016 May 5;11(5):e0154426. doi: 10.1371/journal.pone.0154426 (PMC4858282; doi:10.1371/journal.pone.0154426)
Supplement: S8 Section — (PDF) [file pone.0154426.s008.pdf]

Supporting Information Sections for
Morin, *et al.*, "Nanopore-based target sequence detection"

S8. Detection of 12 bp targets within 5.6 kb dsDNA using size-enhanced γPNA probes

Reagents/Methods for multi-label γPNA binding to long dsDNA

We use a 5.6 kb dsDNA scaffold that is engineered to bind 12-mer γPNA molecules, with each PNA having 3 biotinylated sites for binding avidin (i.e., neutravidin, or streptavidin). The scaffold has 25 distinct sites (Fig. S15) that localizes up to 75 avidin biomarker binding sites. The DNA molecule was synthesized and cloned into a pUC57 plasmid containing kanamycin resistance (Genewiz) and later linearized using the restriction endonuclease ClaI (NEB) prior to PNA invasion.

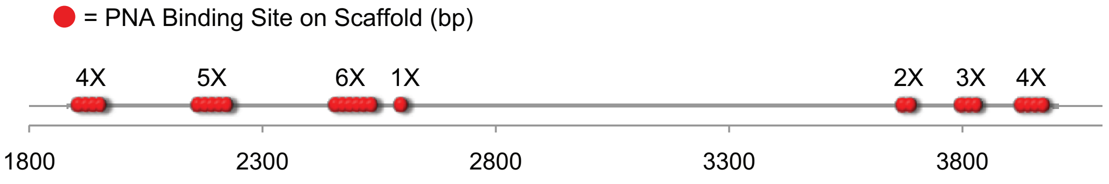

Figure S15: **Schematic of the 25 γPNA binding sites on the 5.6 kb dsDNA scaffold.**

The 12 bp binding γPNA (PNA Bio) has the sequence

GAA*AGT*GAA*AGT

where * indicates the location of a biotin backbone modification that was incorporated via coupling to free amines on a lysine residue.

For the 12 bp binding γPNA study, the 5.6 kbp DNA scaffold was first heated to 95°C for 2 minutes, cooled to 60°C, and then allowed to incubate with a 10-fold excess of γPNA to possible binding sites for 1 hr. Excess γPNA was then dialyzed out of the sample using a 20kDa MWCO dialysis cup (Thermo Scientific). This DNA/γPNA complex was subsequently labeled with a 10-fold excess of neutravidin (Thermo Scientific) to biotin sites. All molecules were stored at -20°C prior to use.

Detection of multiple sites of a sequence in 5.6 kb DNA

The linear 5.6 kbp dsDNA molecule was engineered to contain the 12 bp sequence interspersed at 25 sites within the DNA (Fig. S15). The purpose of this repetition is to boost the sensing signal for each scaffold, since the more occupied PNA sites there are, the longer the nanopore current is impeded, yielding a more easily detected signature. Instead of using bisPNA as the sequence-specific binding molecule, we used the smaller and more versatile γPNA. The work in [1] shows that positive detection and localization of these smaller PNAs is possible, but required a precision sub-4nm pore, and was shown to work with a salt gradient. In the absence of a salt gradient and with a larger pore, we sought to demonstrate positive detection of the presence of the probe by adding bulk to the size of each γPNA. To provide the option of increasing probe size, the γPNA had three biotin molecules incorporated via coupling to free amines on the backbone Lysine amino acid, and using the protein neutravidin as the bulk-adding payload that can bind to each biotin.

After testing complex stability by gel electrophoresis (Fig. S16a), the DNA/γPNA-neutravidin (D/P-N) reagent was tested in a nanopore assay. Figures S16b-d show data comparing δG versus duration distributions for events from three separate experiments conducted on comparable pores. Specifically, DNA alone was tested in an 11 nm pore, neutravidin alone was tested with a 12 nm pore, and the D/P-N reagent was tested with an 11 nm pore, all pores being formed in 10 nm SiN membranes by controlled dielectric breakdown. DNA alone at 2.7 nM yielded 1301 events over 16 minutes, neutravidin alone at 210 nM yielded 2589 events over 11 minutes, and D/P-N complexes at 160 pM yielded 4198 events over 7.3 minutes, with a clear population of fast events present due to a significant amount of free neutravidin as background. Nonetheless, the largest δG events in the D/P-N experiment are attributed to D/P-N complexes (Fig. S16b), providing a simple criteria for tagging events in detecting DNAs that have one or more target 12 bp sequences bound by the γPNA-neutravidin probe.

The mathematical criteria derived can be used to assess confidence in detection. Using the criteria $\delta G > 16$ nS, 390 of the events in the D/P-N experiment are tagged resulting in $Q(p)$ =9.29%. In the prior control experiments, only 0.46% of D and 0.16% of N events are flagged. Though the DNA/γPNA and γPNA-neutravidin controls were not run on this pore, they are unlikely to produce events with $\delta G > 16$ nS, even with folding of the DNA (γPNA create smaller bulges than bisPNA). Regarding false positives, one question is if there are complexes in which neutravidin non-specifically binds to the DNA scaffold; if so, such complexes could cause events with $\delta G > 16$ nS and thus produce a high false positive percentage. The 10X excess neutravidin-to-biotin conditions used do not show an aggregation effect in the presence of γPNA binding to the 25 targets, although aggregation is observed for 36X excess (Fig. S16a). Also, a separate gel showed no visual difference between DNA alone and DNA with 20X excess neutravidin in the absence of γPNA (Fig. S17). These findings suggest that such complexes are unlikely to exist, and therefore would not contribute appreciably to the false positive population. Collectively, these results suggest 0.46% can be used as a (max) false positive probability using the criteria $\delta G > 16$ nS for this nanopore. The 99% confidence test is satisfied, with $Q_* = 1.15\%$ and $Q(p) - Q_* = 8.14\%$ greater than

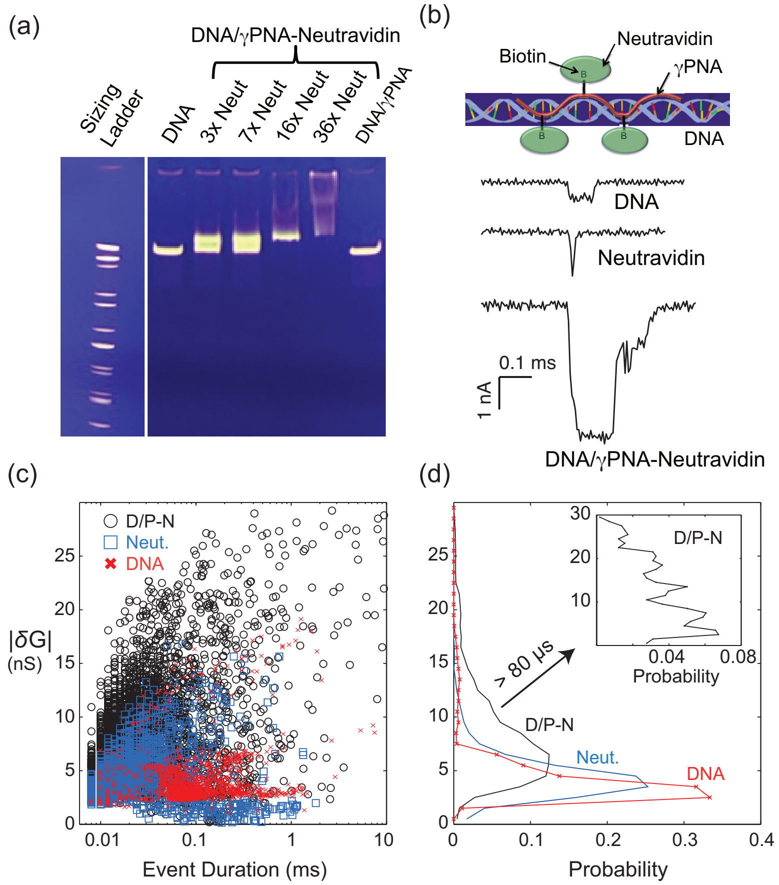

Figure S16: **Positive detection of DNA/(γPNA-biotin)-Neutravidin complexes with an 11-12 nm diameter nanopore.** (a) Image from a DNA/(γPNA-biotin)-Neutravidin (D/P-N) 5% PAGE EMSA using the 5.6 kb DNA scaffold after incubation in recording buffer for 30 mins. The sizing ladder is high molecular weight. The D/P-N lanes contain a 3X, 7X, 16X and 36X excess Neutravidin to biotin. At 36X excess, non-specific binding and/or complex aggregation starts to appear. Nanopore recording was performed at 10X excess neutravidin to biotin to ensure a high percentage of neutravidin-bound biotin sites, while avoiding the aggregation effect observed at 36X in the gel. The DNA/γPNA does not visibly shift compared to the DNA alone. (b) A schematic of one γPNA-biotin-neutravidin region on the 5.6 kb dsDNA scaffold is shown. Representative translocation events are also shown, recorded from consecutive experiments with DNA alone, neutravidin alone, and then D/P-N complexes, all using 11-12 nm pores at 200 mV in 1M KCl. (c) Scatter plot of δG versus duration for the three consecutive experiments (D, N, and D/P-N). (d) Horizontal probability histogram of δG for the three data sets. The inset histogram includes the 578 D/P-N events with duration longer than 0.08 ms, showing considerable spread in the mean δG values and most displaying more than one amplitude level.

$Q(0) = 0.46\%$. The estimated time to results (first time to 99% confidence) is 18 seconds,

exceedingly fast for this data set due to the high capture rate. We note that by exploiting the presence of multiple levels within events [2], which were observed in many D/P-N events including those with $\delta G < 16$ nS, a stronger flagging criteria could be identified to achieve a positive detection result in less time and/or at a lower concentration of molecules.

To test if there are complexes in which neutravidin non-specifically binds to the DNA scaffold, a gel was run and showed no visual difference between DNA alone and DNA with 20X excess neutravidin in the absence of γPNA (Fig. S17). These findings suggest that such complexes are unlikely to exist, and therefore would not contribute appreciably to the false positive population.

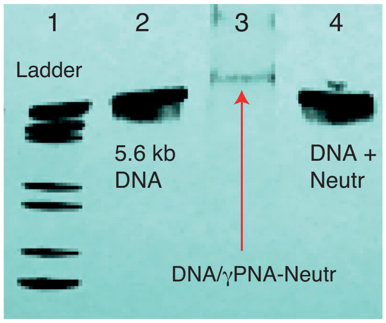

Figure S17: **An EMSA assay shows that excess neutravidin (20X) does not bind non-specifically to the DNA scaffold**. A 4-20% PAGE EMSA comparing: 1) high molecular weight sizing ladder, 2) the 5.6 kb DNA scaffold, 3) DNA/γPNA-neutravidin complex at 10x excess neutravidin to biotin (as in nanopore recording conditions), and 4) DNA with 20x excess neutravidin in the absence of γPNA. Compared to lanes 1 and 2, only 1/10 of the amount was loaded into lane 3, resulting in a band with lower intensity.

References

[1] Alon Singer, Srinivas Rapireddy, Danith H Ly, and Amit Meller. Electronic barcoding of a viral gene at the single-molecule level. *Nano Letters*, 12(3):1722–1728, March 2012.

[2] C Raillon, P Granjon, M Graf, L J Steinbock, and A Radenovic. Fast and automatic processing of multi-level events in nanopore translocation experiments. *Nanoscale*, 4(16):4916, 2012.
