# Supplementary material for: Nanopore-Based Target Sequence Detection
Source: PLoS One. 2016 May 5;11(5):e0154426. doi: 10.1371/journal.pone.0154426 (PMC4858282; doi:10.1371/journal.pone.0154426)
Supplement: S9 Section — (PDF) [file pone.0154426.s009.pdf]

Supporting Information Sections for
Morin, *et al.*, "Nanopore-based target sequence detection"

S9. Details on CFTRΔF508 detection on 300 bp DNA

Nanopore experiments with γPNA were performed in low salt (100 mM LiCl) to increase the stability of the DNA/γPNA complex. An EMSA assay tested incubation of the DNA/γPNA at 250 mM LiCl for 30 min, resulting in 75% of the PNA dissociating from the CFTR sequence. DNA/γPNA complex stability depends on salt in the following ways. First, the presence of high concentrations of cations in 1M LiCl decreases DNA backbone repulsion between bases, and therefore promotes the stability of DNA base pairing. On the other hand, in the absence of salt, PNA exploits backbone repulsion between phosphate groups and is able to displace native bases with its neutral backbone. Unlike the experiment with 25 γPNA sites per DNA performed in high salt (previous section), the CFTR detection has only a single γPNA site per DNA. We therefore used low salt to maximize the likelihood of keeping γPNA in complex with the target-containing DNA.

Since the pore was larger than 20 nm in these results, with a membrane thickness of $L = 20$ nm, we used $d_2(L/2)$ from equation (4) in Section S2 to estimate the nanopore diameters formed by TEM. A description of the nanopore method and devices are in [1]. We used the nanopore-bottle structures described in [1], with the molecules added and captured into the pore from the membrane side of the pore, with the bottle adjacent to the pore on the other side and therefore not playing a role in the event detection and translocation process. In estimating the nanopore size, we use $\sigma = 0.95$ S m^{-1} for 0.1 M LiCl [2, 3].

In a first nanopore 19-20 nm in diameter, 1 nM 300 bp DNA at 200 mV alone produced only 10 detected events over 30 minutes, with most events shorter in duration than the temporal resolution at the bandwidth used (72 μsec, 10 kHz) and only 2 events longer than 100 μsec. The DNA alone events had $\delta G \approx$ 0.1-0.2 nS, and all events were attenuations, consistent with the results for 0.1 M LiCl at 200 mV reported in [4] that also used a 21 nm diameter pore formed by TEM in a 20 nm membrane.

A second nanopore was used to test the DNA/γPNA-PEG (5 kDa) complex, with the γPNA bound to the 22 bp target sequence that comprises the CFTRΔF508 mutation, located centrally within the 300 bp DNA (Methods, main text). The pore was initially 24-25 nm in diameter, and buffer only was first tested producing 3 events over 24 minutes (presumably caused by aperiodic noise). Next with the pore 26-27 nm in diameter, 1 nM DNA/γPNA-PEG was measured at 200 mV produced 46 events over 18 minutes. The majority of events (70%, 32) were longer in duration than the temporal resolution at the bandwidth used (24 μsec, 30 kHz), and 41% longer than 100 μsec. The pore was then enlarged using dielectric

breakdown to 32 nm in diameter, resulting in 30 events over 8 minutes. The pore was enlarged again to 36 nm, resulting in 145 events over 26 minutes. The events produced consistent spread in δG and duration for all three pore sizes, and all events were combined in event plots shown in the main text Figure 6.

The noise characteristics and estimated pore diameter for the three sizes are shown in Figure S18. Since the noise exhibited a peak of 20 pA for the histogram of the open channel current standard deviation (Fig. S18a,b), only events with a mean δG larger than 0.5 nS were included in the event distribution plots shown in the main text Figure 6 (no events met this criteria in the DNA alone control).

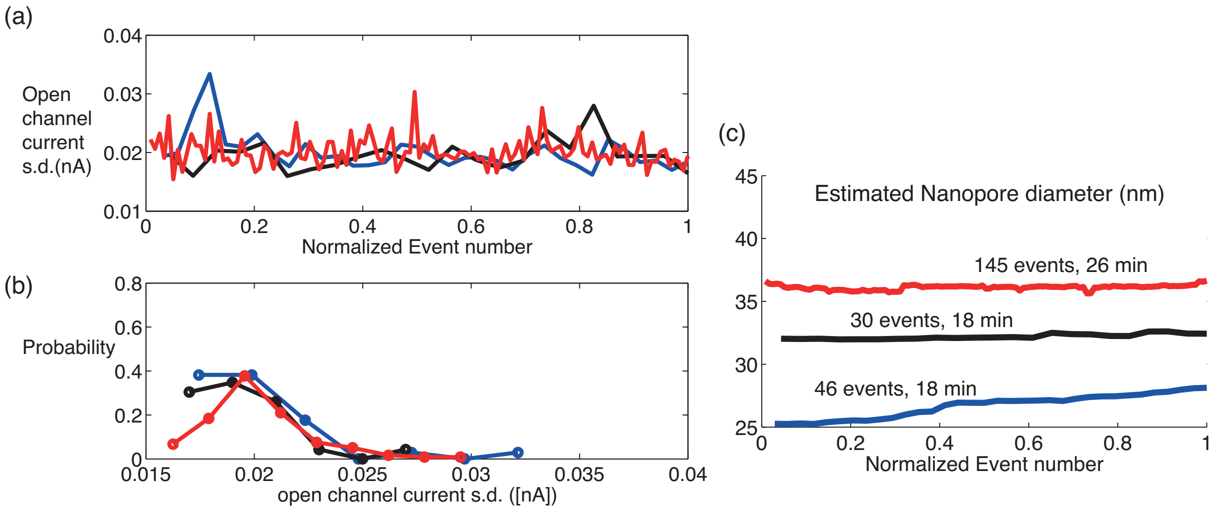

Figure S18: **Noise and estimated diameter of the nanopore during CFTRΔF508 detection assay (Fig 6)**. (a) Evolution of the standard deviation of the open channel current, computed between every pair of events, and (b) corresponding histogram. (c) Estimated nanopore diameter ($d_2(L/2)$) for experiments with 300 bp DNA/γPNA-PEG 5 kDa ($L = 20$ nm membrane, 200 mV, 0.1 M LiCl). The three epochs are separated by pore enlargements using dielectric breakdown.

Representative events comparing DNA alone (20 nm pore) and DNA/γPNA-PEG (26-36 nm pore) are shown in Figure S19.

In addition to the gel images (Fig. 6e), an experiment was performed to further confirm that the full complex DNA/γPNA-PEG produces the deeper and longer lasting events shown in the main text Figure 6 only when the DNA has the CFTRΔF508 mutation sequence. Specifically, a nanopore experiment was performed using the 300 bp DNA containing the wild-type (WT) CFTR gene sequence (methods) after following the same incubation protocol with the γPNA-PEG (5 kDa) reagent as used to form the full complex. The gel image (Fig. 6e) shows conclusively the γPNA-PEG does not bind the WT sequence. Similarly, a nanopore experiment at 100 mM LiCl (200 mV) with the WT 300 bp DNA (1 nM) and γPNA-PEG (1 nM) present did not produce the deeper/longer events shown in Figure 6, but only the faster and shallower events that are consistent with unbound 300 bp DNA alone (Fig. S20).

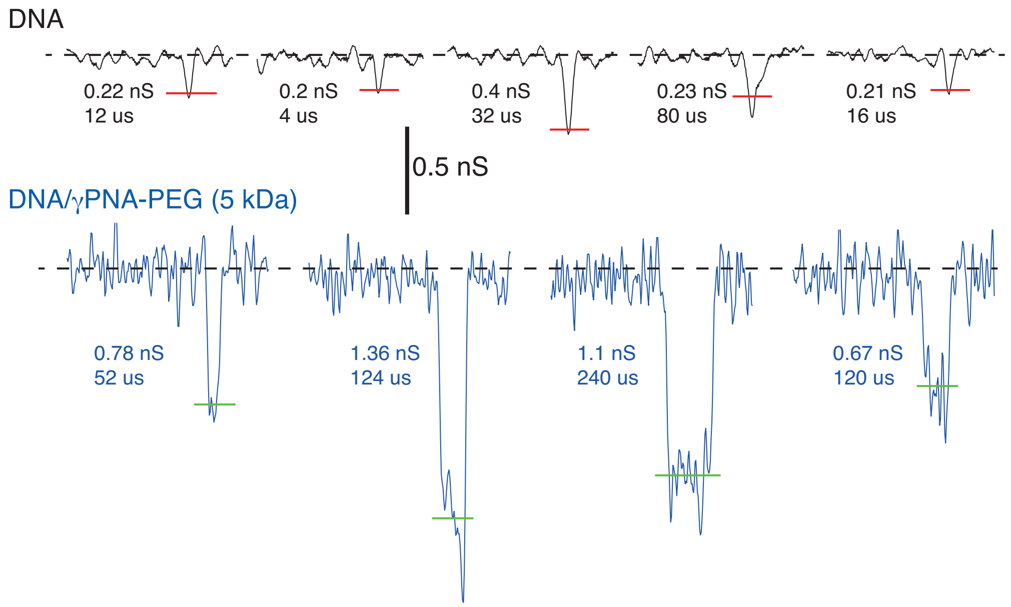

Figure S19: **The CFTRΔF508-positive DNA/γPNA-PEG (5 kDa) complex produced deeper and longer lasting events (bottom) than DNA alone (top).** The 300 bp DNA has γPNA bound to the 22 bp target sequence that comprises the CFTRΔF508 mutation, and the γPNA has a single 5 kDa PEG payload. Representative events report δG and duration values, both at 200 mV in 0.1 M LiCl, with DNA at 10 kHz bandwidth and 19-20 nm pore diameter, and DNA/γPNA-PEG at 30 kHz bandwidth and 26-36 nm pore diameter. A common vertical scale (0.5 nS bar) is shown.

Only 5.2% (6 events out of 115) were longer than 100 μsec. The nanopore was estimated to be 22 nm in diameter and 25 nm in length. Also tested was γPNA-PEG alone (1 nM) which produced almost no events (Fig. S20, red). To show that the pore was functional, the 5.6 kb scaffold (Fig. S15, 0.1 nM) was also tested in higher salt (1M LiCl, 100 mV) with the same pore, and produced the expected event population.

References

[1] Xu Liu, Mirna Mihovilovic Skanata, and Derek Stein. Entropic cages for trapping DNA near a nanopore. *Nature communications*, 6:6222, 2015.

[2] O Sohnel and P Novotny. Densities of Aqueous Solutions of Inorganic Substances. Elsevier, 1985.

[3] A V Wolf. Aqueous solutions and body fluids; their concentrative properties and conversion tables. Hoeber, 1966.

[4] S W Kowalczyk and C Dekker. Salt and Voltage Dependence of the Conductance Blockade Induced by Translocation of DNA and RecA Filaments Through Solid-state

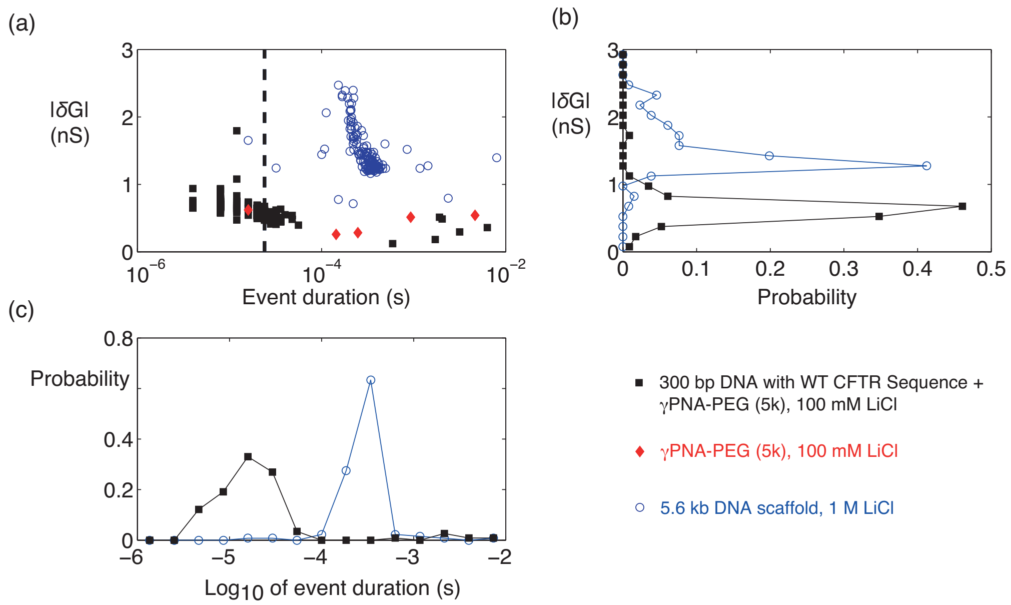

Figure S20: **The CFTR WT 300 bp DNA with γPNA-PEG reagent did not produce deeper/longer lasting events (Fig 6).** Reagents tested include: (i) WT CFTR with γPNA-PEG (1 nM, black), and (ii) γPNA-PEG alone (1 nM, red) in low salt (100 mM LiCl, 200 mV); (iii) 5.6 kb scaffold (0.1 nM, blue) in high salt (1M LiCl, 100 mV). (a) Population of δG vs. duration for (i) 115 events over 22 minutes, (ii) 5 events over 22 minutes, (iii) 131 events over 8 minutes. The pore was 22 nm in diameter for the duration of the experiment. The (b) δG histogram and (c) duration histogram of all events, excluding the γPNA-PEG alone events which were too few.

Nanopores. In *Proceedings of the International Conference on Nanopores for Bioanalytical Applications*, pages 24–31, 2012.
