# Supplementary material for: Nanopore-Based Target Sequence Detection
Source: PLoS One. 2016 May 5;11(5):e0154426. doi: 10.1371/journal.pone.0154426 (PMC4858282; doi:10.1371/journal.pone.0154426)
Supplement: S10 Section — Subsections include details on the Helium Ion Microscope methods for nanopore creation, and a Table that summarizes all experiment conditions. (PDF) [file pone.0154426.s010.pdf]

Supporting Information Sections for
Morin, *et al.*, "Nanopore-based target sequence detection"

S10. Summary of Experimental Conditions

Helium Ion Microscope for nanopore creation

The ORION PLUS microscope is a second generation helium ion microscope (HIM) that features a sub-nanometer image resolution of 0.5 nm. The helium ion beam is based on gas field ion source technology (GFIS). A needle is recursively sharpened in-situ through a proprietary process in which individual atoms are stripped away from the source until an atomic pyramid is created, resulting in only three atoms at the very end of the source tip - a configuration called the "trimer". Once the trimer is formed, the tip is maintained under high vacuum and cryogenic temperatures through a helium gas flow. A high voltage is applied to the needle to produce an extremely high electric field at its apex. The helium gas is attracted to the energized tip where it is ionized. With ionization happening in the vicinity of a single atom, the resulting ion beam appears to be emanating from a region less than an angstrom in size. This produces an extremely bright beam that can be focused to a small probe size. This GFIS source is combined with an advanced electrostatic ion column that focuses the beam with sub-nanometer precision. Much like an SEM, the beam is rastered across the sample, pixel by pixel, for imaging. For nanofabrication, the beam is controlled to execute an user selected pattern with prescribed dosages. The Helium Ion Microscope technical specifications include a resolution of 0.5 nm, beam energy of 10 to 35 kV, and beam current or 0.1 to 100 pA. Experimental conditions for nanopore drilling include the following settings: beam energy 30 kV, beam current 1 pA, and exposure time 15 sec (30 nm SiN membrane).

Summary of Experiment Conditions

The table below presents the varying conditions for the enumerated nanopores employed for the data sets reported. Pores (iii,vii,viii) formed by Controlled Dielectric Breakdown (CDB) were measured with an Axopatch 200B (UofO), while pores (ii,iv-vi, ix, x) used a Multiclamp 700B (TPG).

Table S3: **Summary of Experiment Conditions.**

Pore No.	Figure No.	Pore Formation	Pore diam., length (nm)	LiCl (M)	Voltage (mV)
(i)	1	HIM*	27, 30	1.0	100
(ii)	3, 4, S2-S3	HIM	7, 30	1.0	100, 200
(iii)	4, S3-S5	CDB	6, 10	1.0	200
(iv)	5, S6-S8	HIM*	17-21, 30	1.0	100
(v)	S9-S10	HIM*	36, 30	1.0	100
(vi)	S11-S12	HIM*	50, 30	1.0	90
(vii)	S16†	CDB	11, 10	1.0 (KCl)	200
(viii)	S16‡	CDB	12, 10	1.0 (KCl)	200
(ix)	S16♭	CDB	12, 10	1.0 (KCl)	200
(x)	6, S18-S19	TEM♯	26-36, 30	0.1	200
(xi)	S19†	TEM♯	20, 30	0.1	200
(xii)	6, S20	HIM*	22, 25	0.1, 1	200, 100

* Pore enlarged to the stated size(s) using Controlled Dielectric Breakdown (CDB).

† DNA alone was tested on this pore.

‡ Neutravidin alone was tested on this pore.

♭ DNA/γPNA-neutravidin complex was tested on this pore.

♯ Nanopore formed by TEM as described in [**?**]. Also, the membrane/pore is adjacent to a bottle structure, with reagents added and measured from the opposing side to the bottle.
